# A novel PV power prediction method with TCN-Wpsformer model considering data repair and FCM cluster

**DOI:** 10.1038/s41598-025-95843-9

**Published:** 2025-04-06

**Authors:** Tong Yang, Minan Tang, Hanting Li, Hongjie Wang, Chuntao Rao

**Affiliations:** 1 School of Automation and Electrical Engineering, Lanzhou Jiaotong University, 730070 Lanzhou, China; 2 College of Electrical Engineering, Lanzhou Institute of Technology, 730050 Lanzhou, China

**Keywords:** Photovoltaic power prediction, Data restoration, FCM similar day cluster, Temporal convolutional neural network, TCN-Wpsformer model, Interval forecast, Energy science and technology, Renewable energy, Solar energy, Electrical and electronic engineering, Computer science

## Abstract

Short-term day-ahead photovoltaic power prediction is of great significance for power system dispatch plan formulation. In this work, to improve the accuracy of photovoltaic power prediction, a TCN-Wpsformer (temporal convolutional network-window probability sparse Transformer) day-ahead photovoltaic power prediction model based on combining data restoration and FCM (fuzzy C means) cluster is proposed. The time code of the dataset obtained after data restoration and FCM clustering was spliced with the location code. A temporal convolutional neural network is introduced to extract temporal segment features and incorporate a self-attention mechanism. The short-term photovoltaic power prediction is outputted by the window probability sparse Transformer model in multiple steps. Compared with the original Transformer model, the window probability sparse Transformer model uses the window probability sparse self-attention mechanism. It captures the long-term dependencies while filtering out the time segment features with relatively high importance for computation, which improves the prediction accuracy and reduces the computational cost. The computing time is reduced to 68.83% and R squared is improved by 5.3% compared to Transformer. The comparison is made through 11 models, and the R squared of this model is above 99% while different data volume and different power station data. It proves that the model stability and cross scene generalisation ability is well. Meanwhile, it can also provide more accurate confidence intervals on the basis of point prediction, which has certain application value.

## Introduction

**Fig. 1:**
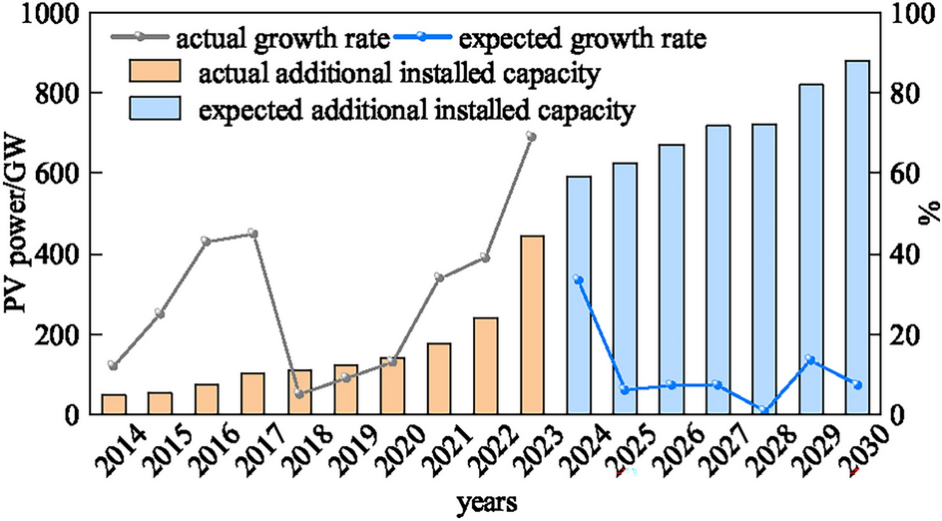
Global newly installed PV capacity schematic.

With the increasing prominence of global warming and environmental pollution, it has become a general consensus of the international community to promote green and low-carbon transformation in the energy sector. It is oriented towards the responsibility to promote the construction of a community of human destiny and the inherent requirement to realize sustainable development^1^. As a major carbon emitter, the power system accelerates the construction of a new type of power system with new energy as the main body. The systematic change of social economy is led through the green and low-carbon development of energy and power, which is conducive to accelerate the realization of clean and low-carbon goals^2^ . As shown in Figure 1, the global growth rate for new installed capacity of solar photovoltaic (PV) systems reaches 69% in 2023^3^. It is predicted that the global installed capacity of this technology may more than triple, reaching a cumulative 2.840 GW by 2030 and 8.519 GW by 2050^4^. Solar PV technology, solar fuel technology, and battery storage technology are among the most prospective technologies in the world. However, PV power generation is characterized by high intermittency and high volatility, and large-scale grid-connected PV brings great challenges to the stable operation of the power grid. Therefore, accurate PV output forecast is crucial to realize its high percentage of elimination and accelerate the construction of a clean, low-carbon, safe and efficient energy system^5^. It is of great significance to build a new generation of power system and make it adapt to the access of high proportion of renewable energy. At the same time, it is of great value to build a comprehensive security defense system of power system and implement risk control^6^.

There are numerous forecast methods for PV power generation, which can be categorized into indirect and direct forecast methods. One of the principles of the indirect prediction method is to predict the PV power generation by using the photoelectric conversion efficiency formula based on the solar irradiance obtained from the calculation^7,8^. However, it is susceptible to the complexity of the solar irradiance calculation model and the time-variant nature of the performance of devices such as inverters. It leads to a cumbersome and poorly interference-resistant molding process with limited practical applications^9^. The direct prediction method is mainly with the help of photovoltaic station output data and meteorological data. And regression analysis and artificial intelligence are used to establish a nonlinear map relationship between influence factors and power generation to directly predict PV power generation^10^. Currently, Markov chain^11^, time series analysis^12^, error backpropagation neural network (BP)^13^ and long short- term memory neural network (LSTM)^14,15^ and so on, which are commonly used direct prediction methods. Although there are many types of prediction methods with different characteristics, the applicability of historical data and the inherent limitations of the algorithms themselves make it difficult for the prediction accuracy to meet the requirements of the power grid.

Therefore, in order to further improve the prediction accuracy, the PV output combination prediction model based on the direct prediction method has received extensive attention from scholars at home and abroad. On the one hand, in order to avoid the model overfitting phenomenon caused by the direct use of massive historical data. Based on the principle of similar day for power load forecasting, the historical days that are highly similar to the elements of the day to be forecasted are selected as training samples. The PV output prediction model based on the similar day theory is constructed^16,17^. For example, Zhou et al^18^ used BP neural network and Extreme Learning Machine (ELM) to establish a PV output prediction model based on the Pearson correlation coefficient method to categorize the weather types and select similar days. Li et al^19^ used grey relation analysis (GRA) to select similar days and established variational mode decomposition (VMD) combined deep limit learning machine PV outflow prediction model. The results show that the prediction accuracy of the combined model is higher compared to a single algorithm. Liu et al^20^ proposed a CNN combined with LSTM for power data prediction model. On the other hand, it is considered that there may be communication interruptions or equipment failures in the process of data collection in PV power plants, which may result in a lack of data for a certain period of time. If this lack of data is not repaired, it affects the power prediction, which in turn affects the normal planning and scheduling of the power grid. Up to now, PV power forecast studies have reported that rainy days perform poorly relative to sunny days^21^ It means that the presence of numerous absent rainfall data values significantly reduces the prediction accuracy. Therefore, there is a need to urgently look at ways to address the lacking data. T Kim et al^22^ compared four methods, linear interpolation (LI), mode interpolation (MI), k nearest neighbor (KNN), and multivariate interpolation for chained equations (MICE), which are used to predict the meteorological and historical PV generation data for solar power generation. Li et al^23^ proposed a generative adversarial network (GAN)-based load data repair model to recover the lacking load data and estimate the baselines of demand response events.

The Transformer model proposed by Google team in 2017^24^ captures the global dependencies of time series data due to the encoder-decoder structure design. The model is already used for a number of spatio-temporal modeling efforts. A short-term prediction of PV power used the Transformer model is presented in the literature^25^. Miguel et al^26^ builds a day-ahead PV power generation prediction model by the application of Time Fusion Transformer (TFT). The multi-head self-attention mechanisms of the Transformer model in the above literature all compute the correlation of time-point features. It lacks the treatment of time-slice features such as photoelectric surge, drop, and violent fluctuation. Tian et al^27^ proposes the sparse self-attention mechanism, which captures time-point features of relatively high importance. However, it neglects the short-term features of the time segments adjacent to the observation point. Liao et al^28^ used a variant model to combine LSTM with Transformer model. The autocorrelation of PV power series and the couple relationship between power and meteorological data are captured to improve the prediction accuracy of PV power series.

**Fig. 2:**
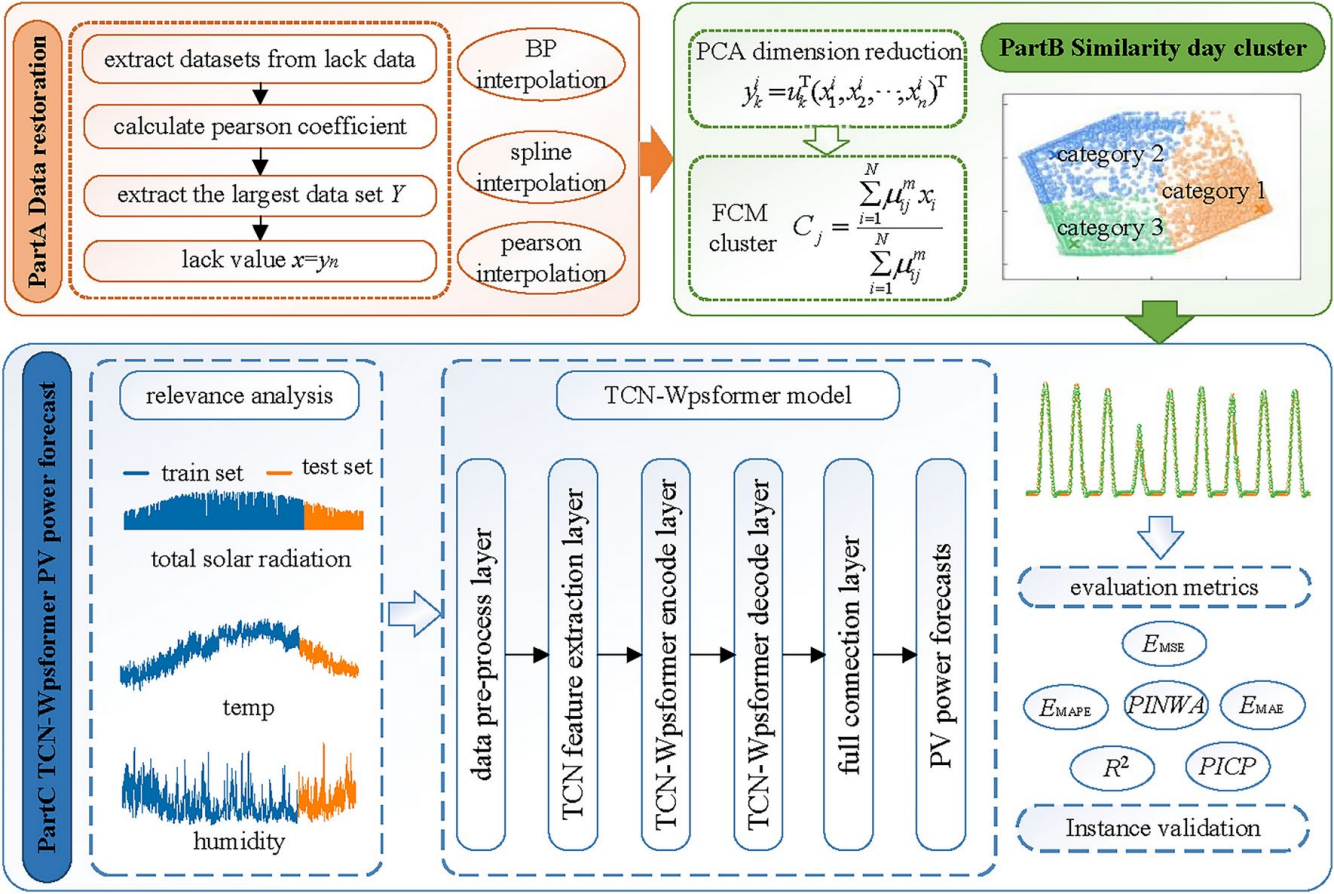
Schematic structure of the proposed method.

Based on this, this study proposes a TCN-Wpsformer day-ahead PV power prediction method that combines data repair and FCM similar-day cluster. The schematic structure of the method is shown in Figure 2. Part A is the PV lack data repair part based on Pearson correlation coefficient method. Part B is the PV power similar day dataset cluster part based on principal component analysis (PCA) degradation and FCM cluster. Part C is the PV power day-ahead prediction part based on TCN-Wpsformer model. Finally, the PV power forecast method proposed is validated by model analysis in a PV power plant in Gansu, China. The results show that the method can realize accurate day-ahead PV power prediction and has certain practical application value.

The main contributions of the proposed research method for photovoltaic power day-ahead prediction combined with data restoration and FCM cluster are as follows.

1. The Pearson correlation coefficient method based PV lack data restoration method has simple steps. The historical data requirement is low and the calculation time is short. It can effectively improve the credibility of data repair.
2. The similar day cluster method based on PCA degradation and FCM is adapted to the prediction of PV power in different weather types. It increases the generalization of the model while improving the prediction accuracy.
3. The window probability sparse multi-head self-attention mechanism is proposed to establish the TCN-Wpsformer model. It incorporates time-point features of higher importance and short-term features of time segments adjacent to the observation point into the self-attention mechanism. It improves the prediction accuracy of PV power and reduces the calculation complexity.

## PV data restoration based on Pearson correlation coefficient

PV power plants require the collection of a large amount and different types of data. These include total radiation, direct radiation, scattered radiation, temperature, humidity, air pressure, wind speed, wind direction and other climatic data, as well as the actual output power. The power station collects data every 5min or 15min, which is a huge amount of data collected every day. However, in the process of data collection, detection and conversion, there may be equipment failure, communication interruption and error code due to extreme changes in weather. It leads to the generation of abnormal data. Therefore, in order to improve the accuracy of day-ahead PV power output prediction, the PV unusual and absent data need to be repaired.

### PV data analysis

**Fig. 3:**
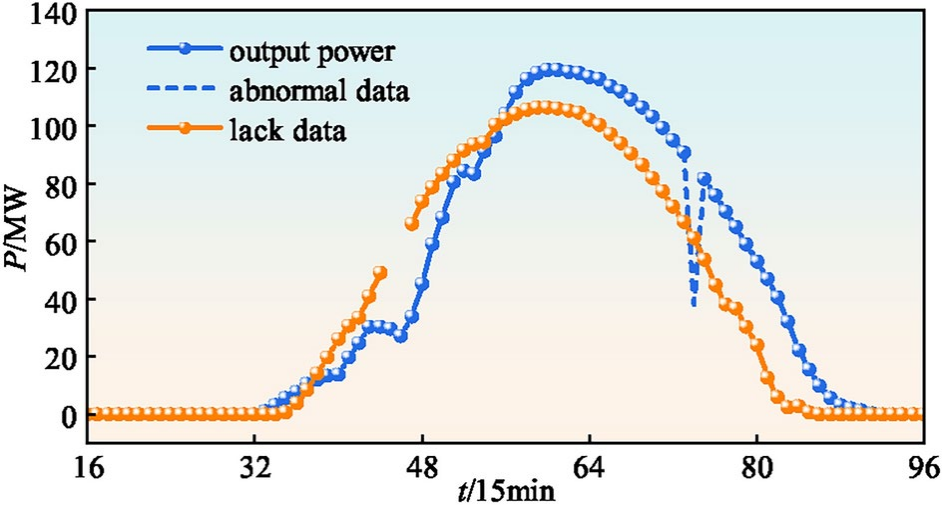
Lanzhou City Regional Road Network Topology.

Abnormal data in power stations mainly includes two types. The first type is the partial absence of data due to equipment failure and communication interruption, as shown in Figure 3. The second type is the occurrence of over-range data or data mutation due to the failure of detection equipment and erroneous code phenomenon. These data often need to be deleted, thus lead to the loss data. Points such as the dotted line in Figure 3 belong to data mutation points.

PV power generation is affected by geographic location, weather, environment and its own module characteristics. The variation of power generation is cyclical and uncertain. Usually, the power generation reaches its maximum value at noon. At the same time, the output power of PV power generation fluctuates with changes in meteorological factors.

### Pearson correlation coefficient data restoration method

Pearson correlation coefficient data restoration method uses $\rho$ to reflect the degree of linear correlation between two variables. The closer the absolute value of $\rho$ is to 1, the greater the correlation between these two variables^29^. In the dataset of this study, there is some kind of linear relationship between the different characteristic variables and the actual power of the PV. The Pearson’s phase relationship can be used to measure the degree of correlation between different characteristic variables and the actual power of PV. Its calculation formula is

1 $$\begin{aligned} {\left\{ \begin{array}{ll} \rho _{{\varvec{{X}}},{\varvec{{Y}}}}=\frac{\textrm{cov}({\varvec{{X}}},{\varvec{{Y}}})}{\sigma _{{\varvec{{X}}}}\sigma _{{\varvec{{Y}}}}}=\frac{n\sum _{i=1}^{n}x_{i}y_{i}-\sum _{i=1}^{n}x_{i}\sum _{i=1}^{n}y_{i}}{\sqrt{n\sum _{i=1}^{n}x_{i}^{2}-\left( \sum _{i=1}^{n}x_{i}\right) ^{2}}\sqrt{n\sum _{i=1}^{n}y_{i}^{2}-\left( \sum _{i=1}^{n}y_{i}\right) ^{2}}} \end{array}\right. } \end{aligned}$$

where, $\textrm{cov}({\varvec{{X}}},{\varvec{{Y}}})$is the covariance of ${\varvec{{X}}}$ and ${\varvec{{Y}}}$. $\sigma _{{\varvec{{X}}}}$ and $\sigma _{{\varvec{{Y}}}}$ are the standard deviation of ${\varvec{{X}}}$ and ${\varvec{{Y}}}$, respectively. Let ${\varvec{{X}}}=\{x_{1},x_{2},\cdots ,x_{n-1},x_{n}\}$ be the data set with one lacking data. Where $x_{1}$, $x_{2}$,..., $x_{n-1}$ are known climate data. *x* is the lack power data to be added.

In the first step, the Pearson correlation coefficients between the climate data ${\varvec{{X}}}_{n-1}=\{x_{1},x_{2},\cdots ,x_{n-1}\}$ of the lacking data set ${\varvec{{X}}}$ and each set of climate data in the complete data set ${\varvec{{Y}}}_{i}$ are calculated separately according to Equation (1). Find the data set ${\varvec{{Y}}}=\{y_{1},y_{2},\cdots ,y_{n-1},y_{n}\}$ that is most similar to climate data ${\varvec{{X}}}_{n-1}=\{x_{1},x_{2},\cdots ,x_{n-1}\}$. Where, $y_{1}$, $y_{2}$,..., $y_{n-1}$ is the climate data most similar to $x_{1}$, $x_{2}$,..., $x_{n-1}$. $y_{n}$ is the known output power.

**Fig. 4:**
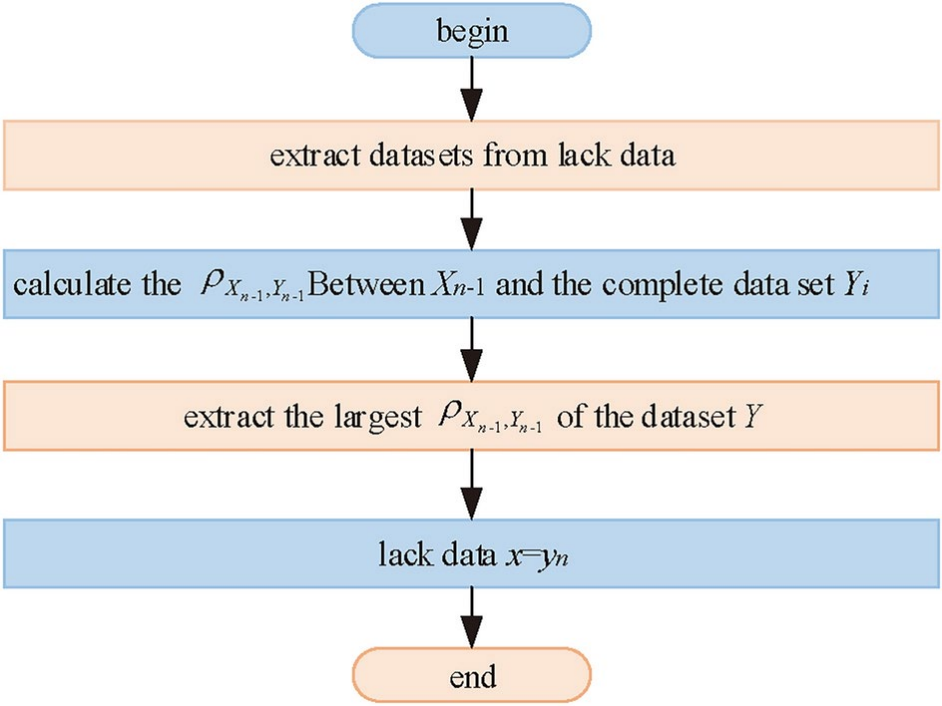
Data restoration flowchart.

The second step takes into account the fact that the output power of the PV plant is closely related to the meteorological data. When the climate data of different moments are similar, the output power of their corresponding moments is also very close. Therefore, it can be directly made *x*=$y_{n}$. That is, the output power of similar moments replaces the output power of lacking moments. The data restoration process based on Pearson correlation coefficient is shown in Figure 4.

The data filled with the method of data restoration is not completely dependent on the data in its neighborhood. The mechanism of output power generation is fully considered. At the same time there is a strong explanatory nature of the data filled. Therefore, it provides higher accuracy for the data repair of fluctuation segments.

## FCM similarity day cluster method

The method that divides different characteristic variables into different categories is cluster. Its purpose is to make the differences be smaller within the same category and larger between different categories. That is, the smaller the differences within groups and the larger the differences between groups, the better effect the cluster gets. FCM is a fuzzy cluster algorithm based on the objective function. It is an improvement of hard cluster algorithm (HCM)^30^. The difference between these two algorithms is that HCM has a hard criterion based on which the data is classified. It leads to a poor classification. Whereas FCM is a flexible fuzzy categorization that puts more emphasis on the degree of affiliation. The higher the degree of affiliation, the more similar it would be.

### PCA dimensionality reduction

PCA is a commonly used data analysis method, and its main idea is to achieve dimensionality reduction by extracting the main components of features^27^. Since the data in this study is different for each feature variable every 15 minutes and the data dimensionality is large. Therefore, PCA is used to normalize and dimensionality reduction of the data. Among the correlations between PV power generation and other feature variables, wind speed, temperature, humidity and irradiance have strong correlations with actual PV power generation, while wind direction and rainfall have negligible correlations with actual PV power generation. In order to improve the convergence of the calculation, the PCA method is used in this study to eliminate the redundant information. The multi-featured variables are transformed into several featured variables to reflect the composite index of the original featured variables. The steps of the method are as follows.

1. Suppose the original data has *m* samples respectively ${\varvec{{X}}}^1$, ${\varvec{{X}}}^2$,...,${\varvec{{X}}}^m$ . Each sample has n-dimensional features ${\varvec{{X}}}^i$=$(x_1^i,x_2^i,..., x_n^i)^\textrm{T}$. Each feature $x_{j}$ has its own eigenvalue. The covariance matrix is derived after normalization operation. If the data is 3-dimensional, the covariance matrix is
2. Obtain the eigenvalue $\lambda$ and the feature vector *u* of the covariance matrix ${\varvec{{C}}}$.
  2 $$\begin{aligned} {\varvec{{C}}}=\begin{pmatrix}\operatorname {cov}(x_1,x_1)& \operatorname {cov}(x_1,x_2)& \operatorname {cov}(x_1,x_3)\\ \operatorname {cov}(x_2,x_1)& \operatorname {cov}(x_2,x_2)& \operatorname {cov}(x_2,x_3)\\ \operatorname {cov}(x_3,x_1)& \operatorname {cov}(x_3,x_2)& \operatorname {cov}(x_3,x_3)\end{pmatrix} \end{aligned}$$
3. The original features are projected onto the selected feature vectors. The new *k* -dimensional feature after dimensionality reduction is obtained with the equation
  3 $$\begin{aligned} {\varvec{{y}}}_k^i={{\varvec{u}}}_k^\textrm{T}(x_1^i,x_2^i,\cdots ,x_n^i)^\textrm{T} \end{aligned}$$

To summarize, for each sample ${\varvec{{X}}}_{i}$, it will be changed from ${\varvec{{X}}}_{i}$ =$(x_1^i,x_2^i,\cdots ,x_n^i)^\textrm{T}$ to ${\varvec{{X}}}_{i}$ =${\varvec{{y}}}_k^i$. In this way, dimensionality reduction can be achieved.

### FCM cluster

The obtained low dimensional data is subjected to FCM cluster. Firstly, the number of clusters and the hyperparameter m are designed. Initialize the affiliation matrix ${\varvec{{U}}}^{(0)}_{n\times m}$=[$u_i^j$] with a random number of [0, 1]. Where *n* is the number of data objects, *m* is the number of clusters, and ${\varvec{{u}}}_i^j$ is the affiliation of data object $x_{i}$ to the cluster center. Then, the cluster center is updated by

4 $$\begin{aligned} C_j=\frac{\sum _{i=1}^N\mu _{ij}^mx_i}{\sum _{i=1}^N\mu _{ij}^m} \end{aligned}$$

Subsequently, the affiliation matrix is updated as shown in Equation (5).

5 $$\begin{aligned} {\varvec{{u}}}_{ij}=\frac{1}{\sum _{k=1}^m\left( \frac{x_i-c_j}{x_i-c_k}\right) ^{\frac{2}{m-1}}} \end{aligned}$$

Finally, it is judged whether the iteration termination condition is satisfied. Under the condition of the maximum number of iterations is not exceeded, the iteration termination condition is

6 $$\begin{aligned} \max _{ij}\left\{ \mid {\varvec{{u}}}_{ij}^{(\textrm{iter})}-{\varvec{{u}}}^{(\textrm{iter})}\mid \right\} \leqslant \varepsilon \end{aligned}$$

## TCN-Wpsformer power forecast model

**Fig. 5:**
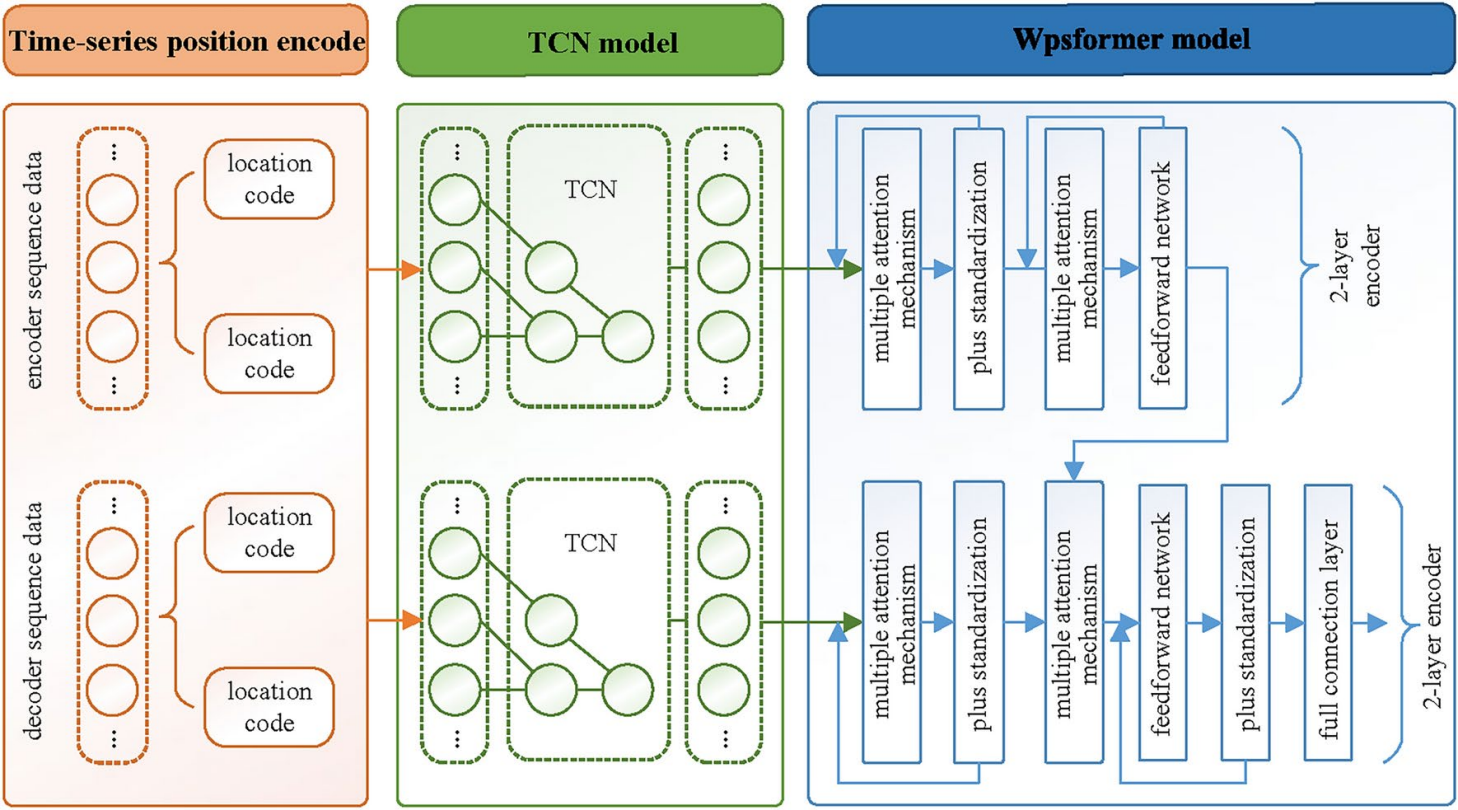
Forecast model based on TCN and Wpsformer.

In this study, a day-ahead PV power forecast model based on MsTCN-Wpsformer is proposed. The model is based on the Transformer model. It consists of independent encoders and decoders. The model network structure is shown in Figure 5.

### Time serial position code

The serial structure of recurrent neural network(RNN) models implies sequential information during model training^31^. While Transformer neural network uses parallel structure framework. It lacks the ability to capture temporal positional features. To compensate this flaw, it often use absolute position encoding to obtain data position information. The encoding formula is

7 $$\begin{aligned} P_{i}^{(2s)}= & \sin (i/10000^{2s/d} \end{aligned}$$

8 $$\begin{aligned} P_{i+1}^{(2s)}= & \cos (i/10000^{2s/d} \end{aligned}$$

where, *i* is the time step. *s* is the number of dimensions. *d* is the data coding of the corresponding position, which takes the value of 512 in this work. The raw data encoded through the position is given sequential coding by the net. Thus, the aim of location information learning is realized. *P* is the absolute location coded data.

### Temporal convolutional network

**Fig. 6:**
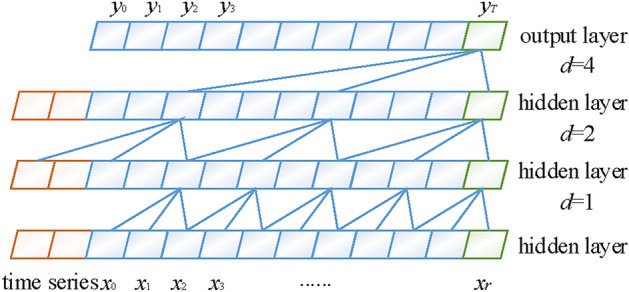
Schematic of DCC structure.

TCN improves both causal convolution and dilated convolution based on the convolution neural network (CNN) model^32,33^. Its dilated causal convolution (DCC) structure is shown in Figure 6. The input time series is set as ${\varvec{{X}}}$ = $x_{1}$,$x_{2}$,...$x_{T}$, and the corresponding output prediction series is ${\varvec{{Y}}}$ = $y_{1}$,$y_{2}$,...$y_{T}$. Where, the *y* value corresponding to a time node is only related to the input data of the current time node and the previous time node. That is

9 $$\begin{aligned} y_t=f(x_1,x_1,\cdots ,x_t) \end{aligned}$$

The above operation eliminates the leakage of future information from the convolution operation and strictly enforces the constraints of the data sequence in the time direction. The DCC allows the inputs to be sampled at intervals during convolution, and the size of the receptive field grows exponentially with the number of layers. Thus, fewer layers can be employed to obtain a larger receptive field. The equation is

10 $$\begin{aligned} H\left( l\right) =\sum _{i=0}^{k-1}h\left( s\right) x_{l-d_ms} \end{aligned}$$

where, *H*(*l*) is the output of the *t*-th neuron of the TCN. *k* is the size of the convolution kernel. *h*(*s*) is the sth element of the convolution kernel. $x _{l-d_{m}s}$ is the input value corresponding to the shift from time *l* to time $d_{m}s$. $d_{m}$ is the dilation factor. The valid history information that can be extracted by one layer is (*k*-1)*d*.

In the case of PV power anomalies or changes, TCN can expand the sensory field of the model by additional layers. Further, more data features are extracted. In addition, TCN is introduced to improve the inherent shortcomings of the Transformer-like model.

### Window probability sparse transformer

The PV power data is processed by TCN and then entered into Wpsformer model. The multi-head attention mechanism of Transformer model has problems such as high computational complexity and low speed due to the long time sequence data. The window probability sparse multi-head self-attention mechanism is proposed. Through the local attention mechanism of fixed window, the relatively important probabilistic sparse self-attention mechanism is filtered and then calculated. Through the local attention mechanism of fixed window, the relatively important probabilistic sparse self-attention mechanism is filtered. In order to achieve the purpose to reduce calculation complexity and ensure precision.

#### Multiple attention mechanism

**Fig. 7:**
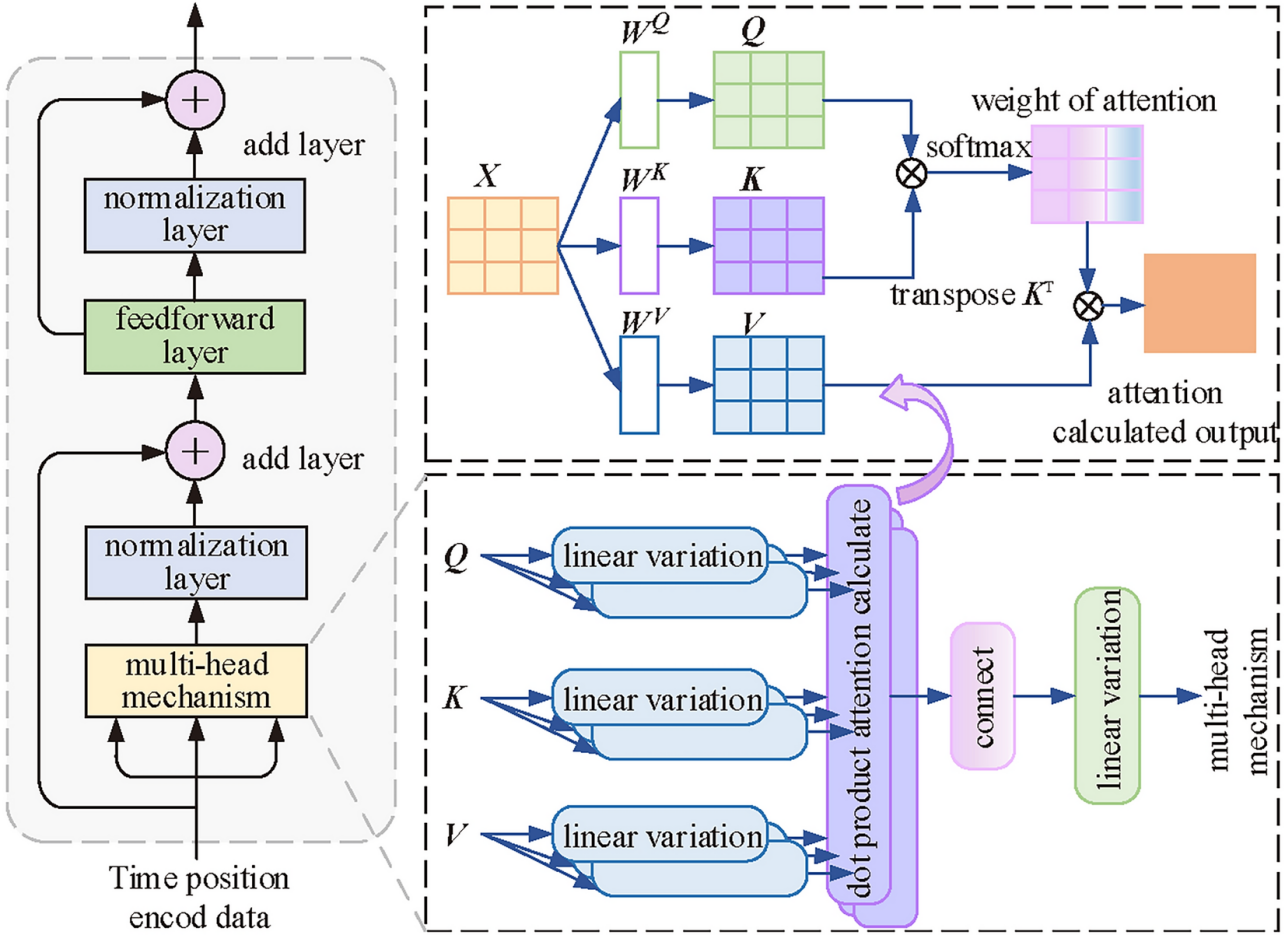
Calculation process of multi-head attention mechanism in Transformer.

The core part of the traditional Transformer encoder is the multi-head attention mechanism^34^. It consists of multiple heads with self-attention. The process can be described as a process solved by a query vector and a set of key-value vector matrices. The principle is shown in Figure 7.

In Figure 7, the query vector ***Q***, the key vector ***K***, and the weight vector ***V*** are transformed from the previous output. In the actual operation, the model computes the attention function on a set of query vectors simultaneously. It packs them into matrix ***Q***. And packs the key vector and the value vector into matrices ***K*** and ***V***. Matrices ***K*** and ***V*** are assigned the same value as ***Q*** in self-attention^35^. The result of the output calculation is the weighted value of the weights. The weight is derived from the compatibility operation between the query vector and the key vector. ${\varvec{{f}}}=\{f_i\}_{i=1}^t$ is the input to the multi-head self-attention module. ***K***, ***V*** and ***Q*** are computed as

11 $$\begin{aligned} {\left\{ \begin{array}{ll}\varvec{K}_j=\varvec{fW}_j^k\\ \varvec{V}_j=\varvec{fW}_j^\nu \\ \varvec{Q}_j=\varvec{fW}_j^q\end{array}\right. } \end{aligned}$$

where, ${\varvec{{W}}}_j^k,{\varvec{{W}}}_j^\nu ,{\varvec{{W}}}_j^q\in \textbf{R}^{d\times d_k}$is the trainable projection matrix. With the export results, the scaled dot product attention calculation is carried out, which is given by

12 $$\begin{aligned} \textrm{Attention}(\varvec{Q},\varvec{K},\varvec{V})=\textrm{softmax}(\frac{\varvec{Q}_j\varvec{K}_j^\mathrm {~T}}{\sqrt{d_k}})\varvec{V}_j \end{aligned}$$

In order to jointly attend to the information from different representational subspaces at different locations, further optimization is required. *H* parallel attentional computations can be used. In case of ${\varvec{{W}}}^A\in \textbf{R}^{Hd_k\times d}$ , the multi-head attention mechanism is computed as

13 $$\begin{aligned} \textrm{MultiHead}({\varvec{{Q}}},{\varvec{{K}}},{\varvec{{V}}})=\textrm{Concat}\left( \left\{ head_j\right\} _{j=1}^H\right) \varvec{W}^A \end{aligned}$$

The complexity of the mechanism increases exponentially with the sequence length. The window probability sparse self-attention mechanism model proposed in this study is described in detail below.

#### Window probability sparse self-attention mechanism

**Fig. 8:**
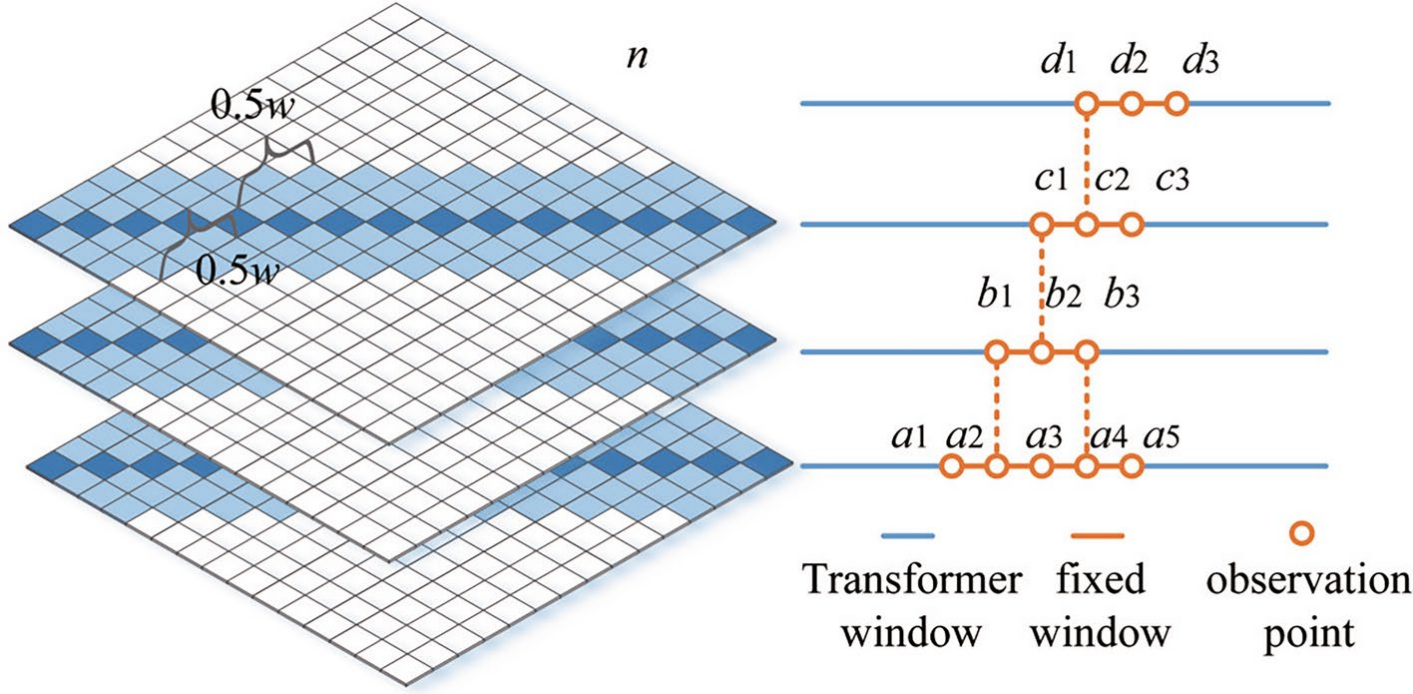
Window sparse self-attention mechanism model.

The self-attention matrix of the window sparse self-attention mechanism is shown in Figure 8. Where *n* is the length of the input sequence. *w* is the window size. $a_{1}$ $\sim$ $a_{5}$, $b_{1}$ $\sim$ $b_{3}$, $c_{1}$ $\sim$ $c_{3}$, $d_{1}$ $\sim$ $d_{3}$ are the observation points. In the field of ultra-short-term wind power prediction, the data closer to the prediction point are more important compared to the observation points farther away from the prediction point. The correlation between any nine observation points needs to be calculated in the original Transformer model. In contrast, in the matrix of Figure 8, only the observation points near the fixed window are processed with the local attention mechanism. The white part outside the window is filled with zero vectors. The computational complexity of the original Transformer is $O(n^{2})$. Increasing exponentially with the sequence length *n*, the computational complexity of the window sparse self-attention is just *O*(*nw*) .

For a windowed sparse self-attention mechanism with l layers, the topmost layer has a receptive field of *lw* with *w* fixed. The range covered by the attention of observation point $a_{2}$ in layer 1 in Figure 8 is $a_{1}$ $\sim$ $a_{3}$, and the window size is *w*. The range covered by the attention of observation point $b_{2}$ in layer 2 is $b_{1}$ $\sim$ $b_{3}$, and the window size is also *w*. And the observation point $b_{1}$ in layer 2 contains the local information captured at observation point $a_{2}$. Observation point $b_{3}$ contains the local information captured at observation point $a_{4}$. Observation point $b_{2}$ is able to use all the information of observation points $a_{1}$ $\sim$ $a_{5}$. Under the window sparse self-attention mechanism, although the bottom layer only captures local information, the top layer’s sensory field expands as the number of layer increases.

Finally, the above proposed window sparse self-attention matrix and probabilistic sparse self-attention matrix are spliced and superimposed to form the window probabilistic sparse self-attention mechanism model shown in Figure 8. The white part of it is filled with zero vectors. The improved window probabilistic sparse self-attention mechanism function is

14 $$\begin{aligned} A(\varvec{Q},\varvec{K},\varvec{V})=\textrm{softmax}(\frac{\overline{\varvec{Q}}_1\varvec{K}^\textrm{T}+\overline{\varvec{Q}}_2\varvec{K}^\textrm{T}}{\sqrt{d}})\varvec{V} \end{aligned}$$

where, $\overline{\varvec{Q}}_1$ and $\overline{\varvec{Q}}_2$ are sparse matrices with the same dimension as the self-attention matrix in the original Transformer model. The final Wpsformer model incorporates both highly important time-point features and observation proximity features into the self-attention mechanism, which reduces the computational complexity.

#### Feed-forward neural network

In this study, the matrix is processed by layer normalization and residual linkage. Layer normalization and residual links can solve the problems of gradient loss and network degradation caused by too many layers in deep neural networks to a certain extent. It can also accelerate the convergence of the model prediction results. The formula of Feed-forward neural network (FNN)^36^is

15 $$\begin{aligned} F\begin{pmatrix}k\end{pmatrix}=\max \begin{pmatrix}0,kW_1+b_1\end{pmatrix}W_2+b_2 \end{aligned}$$

where, *F*( ) is the feed forward neural network function. *k* is the output value of the normalization layer. ${{\varvec{W}}}_{1}$ and ${\varvec{{b}}}_{1}$ are the weight matrix and bias vector of the 1st linear layer respectively. ${\varvec{{W}}}_{2}$ and ${\varvec{{b}}}_{2}$ are the weight matrix and bias vector of the 2nd linear layer respectively.

Ultimately, the TCN-Wpsformer model outputs forecasts in a single multi-step output that uses a fully-connected layer.

### Day-ahead PV power forecast

**Fig. 9:**
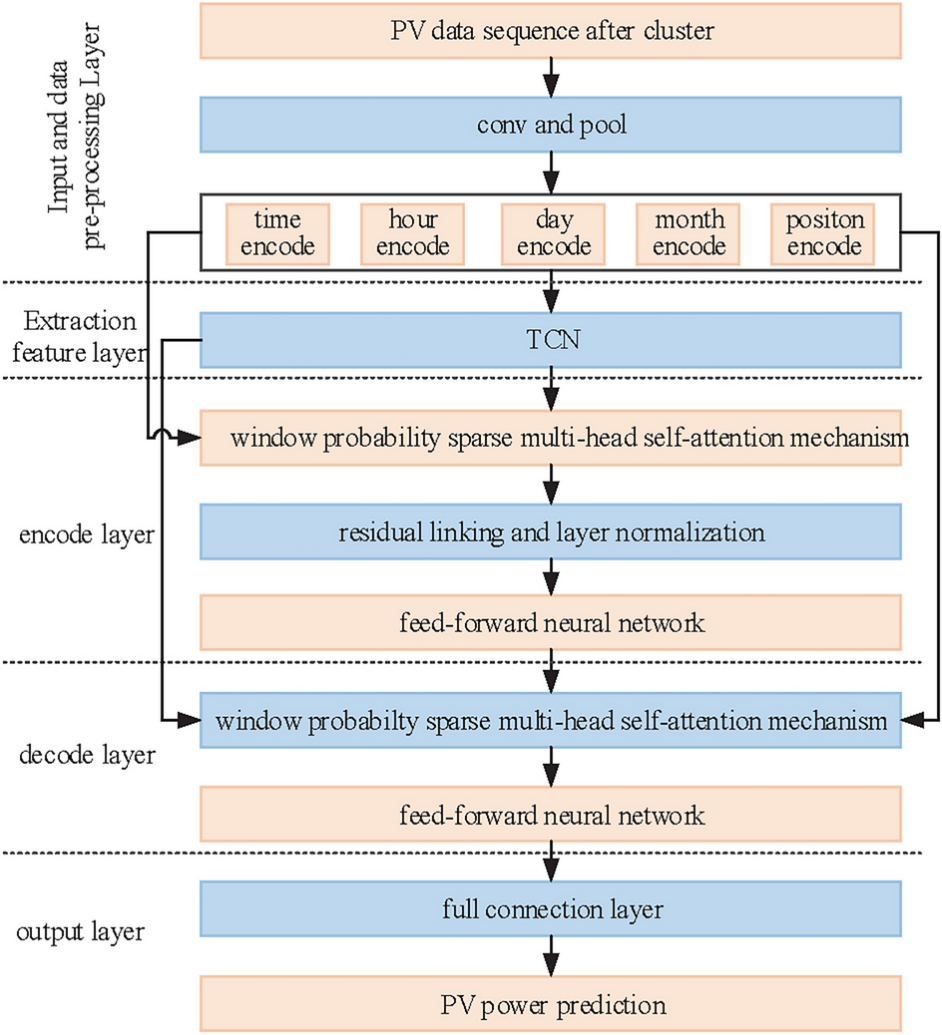
PV power day-ahead prediction flow.

Based on the above TCN-Wpsformer hybrid model structure. The PV power short-term prediction process is shown in Figure 9. The specific steps are as follows.

1. The original data of the photoelectric field is fed into a convolution pooling layer for dimension mapping to obtain a new time series matrix. Then the time features are dimensionally transformed by a 1D convolutional layer. The converted time feature matrix is spliced with the position encoding matrix.
2. Feature extraction of the spliced matrix is performed with TCN to capture the long-term dependencies between the spliced matrices.
3. Input the output matrix of the TCN and the splice matrix into the coding layer. Transform the TCN output matrix into query vectors and key vectors by weights matrices ${\varvec{{W}}}^{{\varvec{{Q}}}}$ and ${\varvec{{W}}}^{{\varvec{{K}}}}$ which are required to compute the attention. The splice matrix is to be transformed into value vectors through the weight matrix ${\varvec{{W}}}^{{\varvec{{V}}}}$. The matrix ${\varvec{{Q}}}$, ${{\varvec{K}}}$ and ${\varvec{{V}}}$ are obtained after the attention mechanism function. The intermediate feature matrix is obtained through residual network, normalization process and feed forward neural network which is available in decode layer.
4. The masked spliced matrix is fed into the TCN. The output matrix of TCN together with the masked spliced matrix enters the decoding layer. The query vector is obtained by masking window sparse multi-head self-attention mechanism and residual join and normalization process.
5. The key vector and value vector obtained through the decoding layer. The key vector, value vector and query vector together go to the next sub-layer of the decoder. Finally the PV power forecast is obtained through the fully connected layer.

## Experiment and discussion

The superiority of the proposed model in this study is mainly verified through five parts of experiments. It includes data restoration experiments, different cluster methods comparison, TCN-Wpsformer model prediction comparison, ablation experiments as well as model complexity and interpretability.

### Correlation analysis of dataset and evaluation indicators

**Fig. 10:**
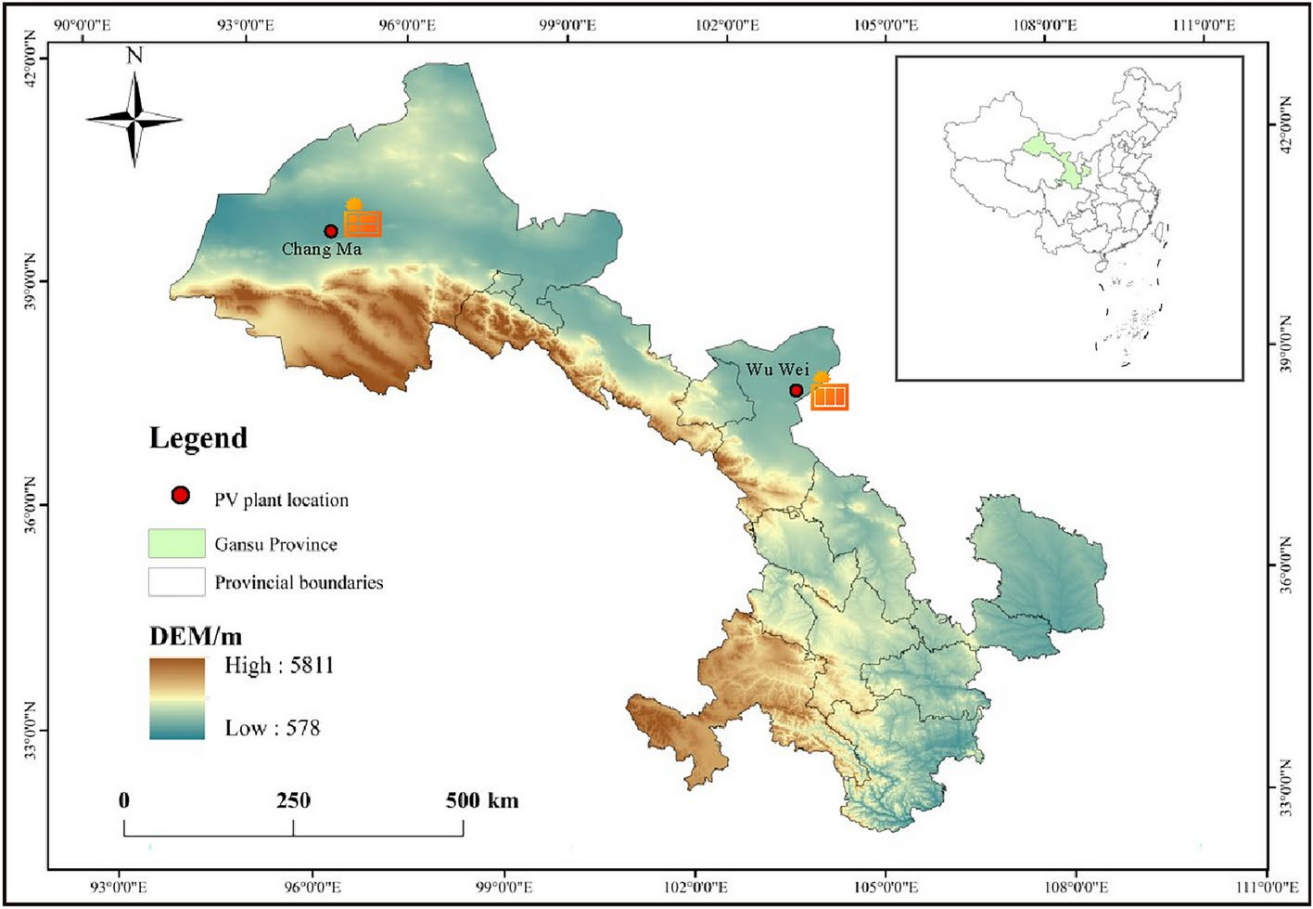
Geographic location of the photovoltaic plant.

The experimental data in this work is derived from PV power data from a PV plant in Gansu, China, and data from the local meteorological bureau. The geographic location of this PV plant is shown in Figure 10. It is includes Changma PV plant and Wuwei PV plant. The experimental dataset is selected from the PV power data for a whole year from January 1, 2021 to December 31, 2021, sampled every 15 min. It also includes the remaining elements as radiation index, scattering index, total horizontal radiation, azimuth, precipitation, humidity, wind speed, wind direction, temperature and power, respectively. During the experiment, the dataset is divided into train and test sets in a ratio of 3:1, through which the train set is used to train and build the model and the test set is used to evaluate the prediction ability of the model.

**Fig. 11:**
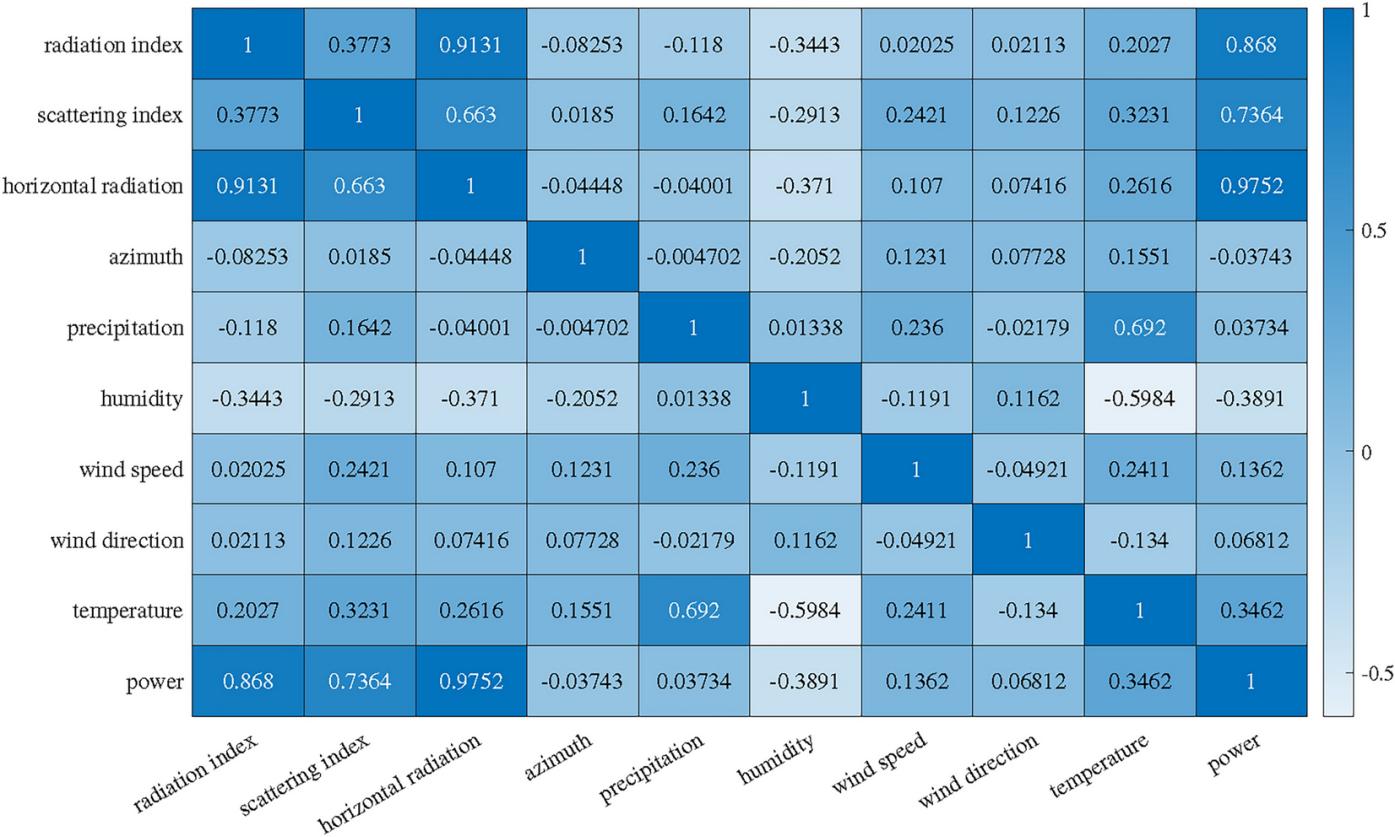
Correlation analysis results.

In the dataset of this work, the correlation and the degree of correlation between different feature quantities and PV power can be measured by the Perason correlation coefficient shown in Equation (1)^37^. The results are shown in Figure 11.

From Figure 11, it can be clearly seen that the radiation index, scattering index, total horizontal radiation, humidity and temperature have more influence on the actual power. Based on the correlation results, the 6-dimensional data with the highest correlation is selected as the input characteristic parameter for subsequent PV power prediction experiments.

The data normalization is a common data processing method. It enables the data to achieve convergence to a certain extent, thus reduces the problem of gradient explosion caused by the prediction process. The normalization method used in this study is the 0 to 1 normalization method which is shown in Equation (16).

16 $$\begin{aligned} x^*=\frac{x-x_{\min }}{x_{\max }-x_{\min }} \end{aligned}$$

Where, $x^*$ is the normalized data. *x* is the actual data value. $x_{\min }$ and $x_{\max }$ are the minimum and maximum values in the original data set, respectively.It reduces the probability of gradient explosion while still retains the complete feature trend. It possesses better trainability, so the normalized data is chosen as the network input.

In order to quantitatively evaluate the prediction accuracy of different models, this study uses four evaluation indexes to compare the models. Namely, the mean square error (MSE), mean absolute error (MAE), mean absolute percentage error (MAPE) and R squared (R2). Among them, MSE and MAE evaluate the prediction accuracy from the perspective of the size of the error, and MAPE measures the average percentage size of the error, the smaller the three values indicate a more effective forecast.^38^. $R^{2}$ indicates the extent to which the model explains the changes in the dependent variable or measures how well the model fits the observations^39^. The larger the value, the better the prediction. The four indicators are calculated as

17 $$\begin{aligned} E_{\textrm{MSE}}= & \frac{1}{n}\sum _{i=1}^{n}(\hat{y}_{i}-y_{i})^{2} \end{aligned}$$

18 $$\begin{aligned} E_{\textrm{MAE}}= & \frac{1}{n}\sum _{i=1}^{n}\mid \hat{y}_{i}-y_{i}| \end{aligned}$$

19 $$\begin{aligned} E_{\textrm{MAPE}}= & \frac{1}{n}\sum _{i=1}^n|\frac{\hat{y}_i-y_i}{y_i}|\times 100\% \end{aligned}$$

20 $$\begin{aligned} R^{2}= & 1-\frac{\sum _{i=1}^N(y_i-\hat{y}_i)^2}{\sum _{i=1}^N(y_i-\overline{y_i})^2} \end{aligned}$$

where, $\hat{y}_{i}$is the predicted value of model at moment *t*. $y_{i}$ is the true value at moment *t*.

### Data restoration experiment

The entire data is divided into dataset A and dataset B. Dataset A is the dataset to be restored. It is only meteorological data for each sample in dataset A, which does not contain actual power values. The data for each sample point is of the form ${\varvec{{X}}}_{i}$=[$x_{1}$, $x_{2}$, $x_{3}$, $x_{4}$, $x_{5}$], which are radiation index, scattering index, total horizontal radiation, humidity and temperature, respectively. The data for each sample point in dataset B is of the form ${\varvec{{Y}}}_{i}$=[$y_{1}$, $y_{2}$, $y_{3}$, $y_{4}$, $y_{5}$, $y_{p}$] , which are radiation index, scattering index, total horizontal radiation, humidity, temperature, and actual power values, respectively. To verify the accuracy of Pearson’s correlation coefficient interpolation. A period of continuous time is selected randomly in dataset A and its actual power value is artificially removed. Then follow the flowchart in Figure 4 to restore the data.

Firstly, take a sample point${\varvec{{X}}}_{i}$=[$x_{1}$, $x_{2}$, $x_{3}$, $x_{4}$, $x_{5}$] in the dataset A to be repaired. The correlation coefficients between ${\varvec{{X}}}_{i}$ and each set of meteorological data ${\varvec{{Y}}}_{i}$=[$y_{1}$, $y_{2}$, $y_{3}$, $y_{4}$, $y_{5}$, $y_{p}$], *j*=1,...,*n* in the complete dataset B are calculated separately, as shown in Equation (20).

21 $$\begin{aligned} \rho _{X_i,Y_i}=\frac{6\sum _{n=1}^6x_{in}y_{in}-\sum _{n=1}^6x_{in}\sum _{n=1}^6y_{in}}{\sqrt{6\sum _{n=1}^6y_{in}^2-(\sum _{n=1}^6y_{in})^2}\sqrt{6\sum _{n=1}^6x_{in}^2-(\sum _{n=1}^6x_{in})^2}} \end{aligned}$$

Take ***Y*** which maximises the correlation coefficient $\rho _{X_i,Y_i}$ . Let $Y^{'}=Y\mid _{\rho =\max \{\rho _{X_i,X_i}\}},\quad j=1,\cdots ,n$ . The actual power $y_{p}$ in the sample point be used as the actual power $x_{p}$ of ${\varvec{{X}}}_{i}$ in the dataset A. That is, the actual power in the complete data set with the largest correlation coefficient with ${\varvec{{X}}}_{i}$ is taken in place of the lacking power of ${\varvec{{X}}}_{i}$.

The spline interpolation and BP interpolation are chosen as comparison experiments. The data after interpolation of cubic spline interpolation method can be obtained by using the internal function interpolate in python. The BP neural network parameters are configured by combining data characteristics and task complexity, which is set as shown in Table 1.

**Table 1:** BP neural network parameters configuration basis.

Parameters	Value	Rationale
Num neurons in the input layer/pc	6	Input data dimensions after correlation analysis are 6 dimensions
Num neurons in the output layer/pc	1	Output layer set to 1 when repairing a single vacant power value
Num of hidden layers/pc	1	Task complexity is moderate, in order to avoid overfitting
Num of neurons in hidden layer/pc	8	Number of nodes from 2 to 11, lowest MSE indicator at 8
Learning rate	0.01	Use Adam optimiser, learning rate 0.01 usually performs consistently

The input layer inputs the six feature quantities after dimensionality reduction. The output layer is the PV power. The complete historical dataset is used as training data. Afterwards, the feature quantities of the data to be repaired are inputted to obtain the repaired values through the BP model.

**Fig. 12:**
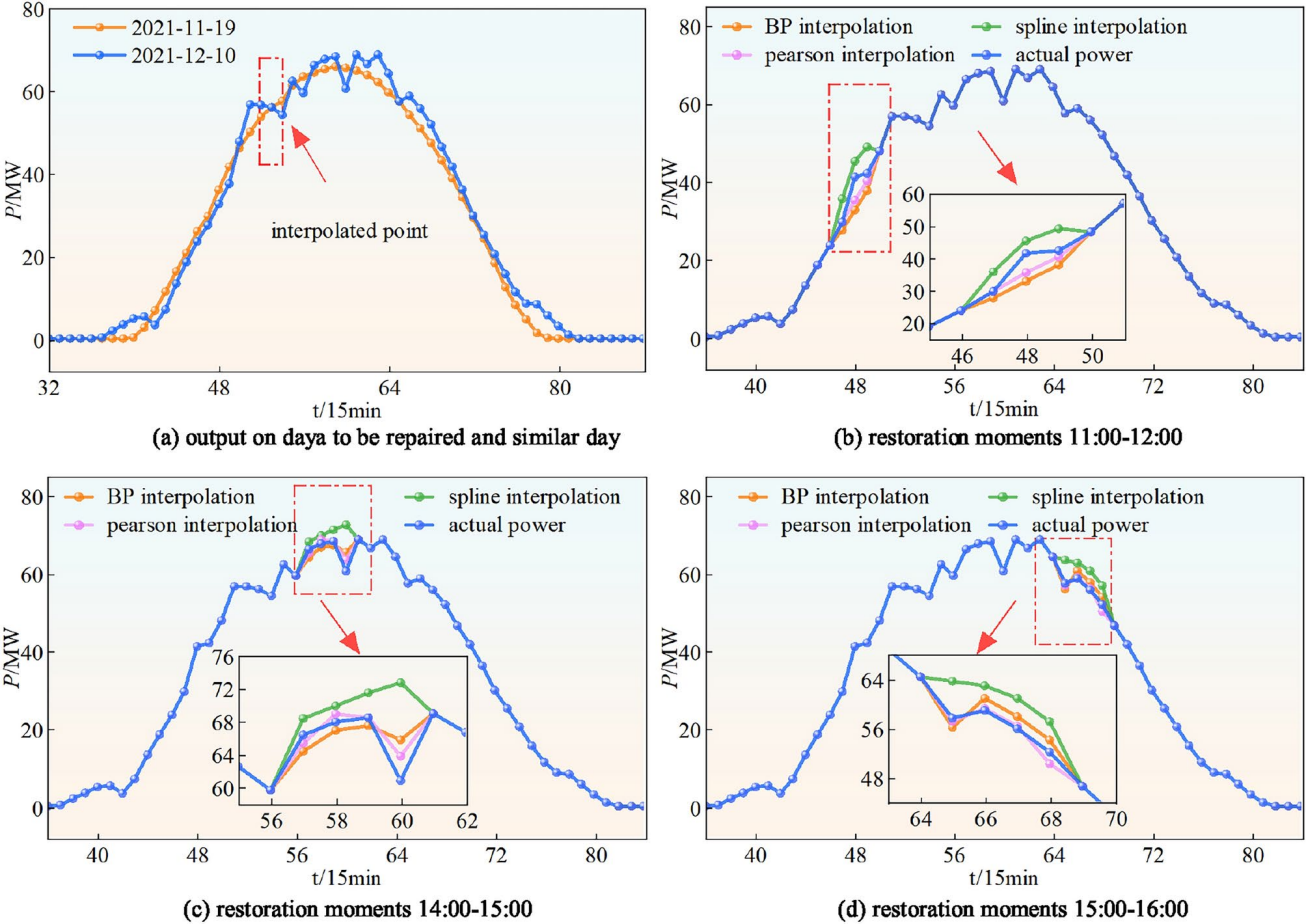
Comparison of data restoration results at different moments.

The validation experiment is for the same length of lack data at different time periods. The data is repaired with cubic spline interpolation and Pearson’s correlation coefficient interpolation in the case of time period 11:00-12:00, 14:00-15:00, and 15:00-16:00 , respectively. Take the 14:00 sample point repair as an example, the most similar moment point to the 14:00 moment in 2021.12.10 is calculated in dataset B to be the 14:000 moment point in 2021.11.19. Therefore, the actual power of the 14:00 moment point in 2021.11.19 is made to be the restoration power of the 14:00 moment in 2021.12.10. The data repair result curve is shown in Figure12. Considering that PV system power generation is affected by environmental parameters such as irradiance and temperature. The data errors are often associated with physical factors. The device failure often manifests itself as an abnormal deviation in a specific direction. Therefore, the average error (mean error) and the MAE are chosen to be used as the evaluation indexes , whose results are shown in Table 2.

**Table 2:** Comparison of data restoration results by different methods/MW.

Method	Index	Time period		
		11:00-12:00	14:00-15:00	15:00-16:00
Spline interpolation	ME	3.58	6.25	3.79
	MAE	3.96	7.04	5.68
BP interpolation	ME	-2.56	-3.07	2.62
	MAE	2.77	4.24	2.95
Peason interpolation	ME	-1.27	2.62	1.36
	MAE	1.88	3.50	2.45

As it can be seen that the data restoration based on Pearson’s correlation coefficient in any time period is better than data restoration by cubic spline interpolation and BP interpolation. The method not only improves the accuracy of data restoration, but also maximizes the use of the residual data.

### PCA-FCM data cluster experiment

PCA downscaled eigenvalue $\lambda$ = [2.0976, 0.5532, 0.4061, 0.0721, 0.0354, 0.0066]. Its cumulative contribution can reach 98.677% when *k*=4. Therefore, the number of principal components retained after PCA dimensionality reduction is chosen as 4 in this study.

**Fig. 13:**
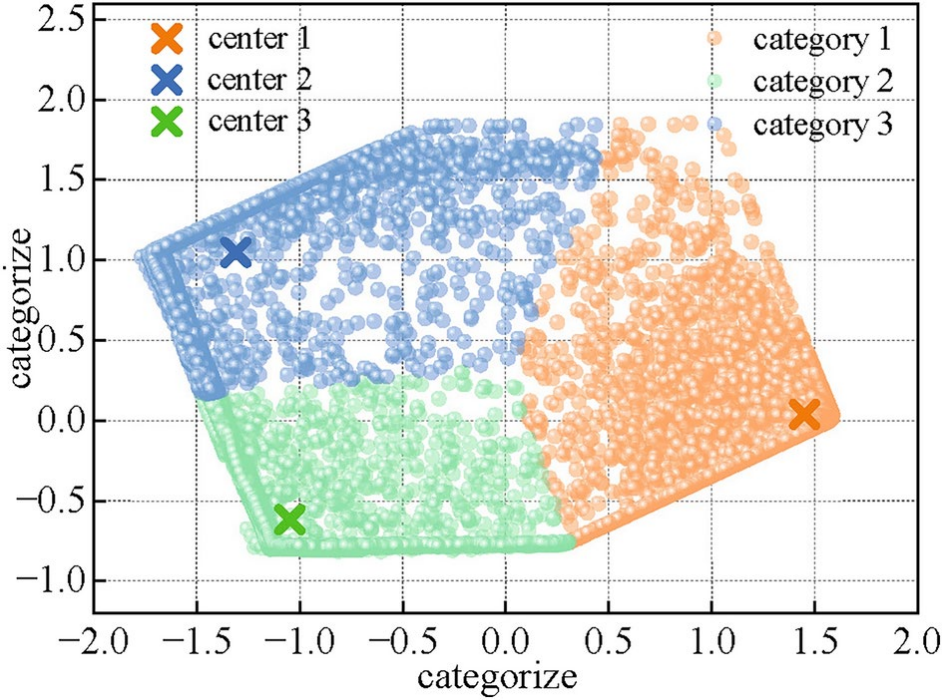
Similar day cluster results of FCM.

**Fig. 14:**
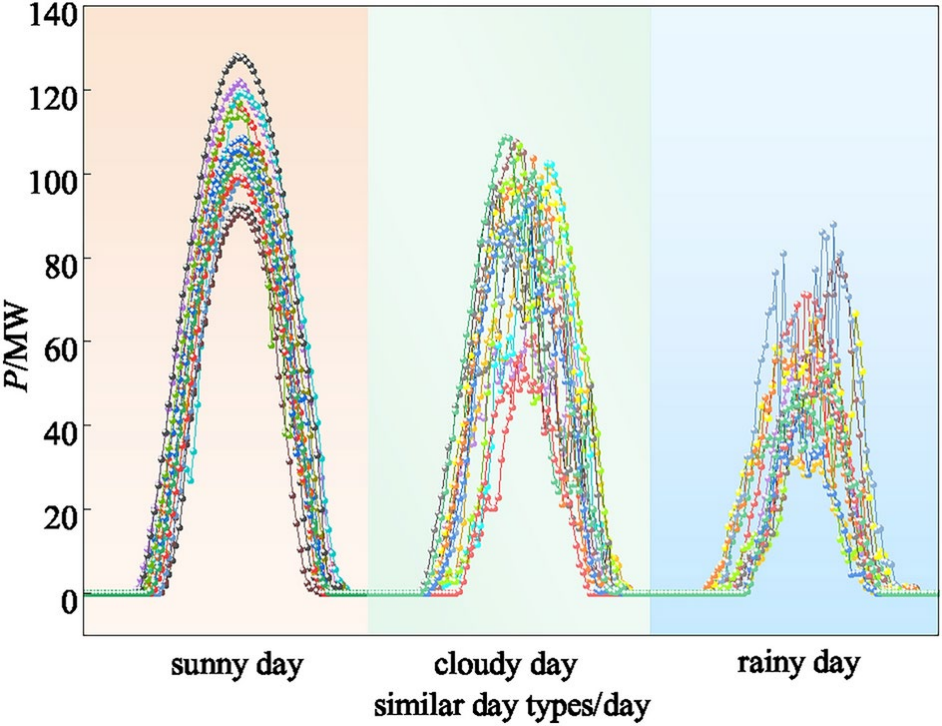
Sequence of PV power output on different types of similar days.

The FCM similar day cluster visualization is shown in Figure 13. The data set is divided into three categories by weather types which are cloudy, sunny and rainy based with the cluster results. The specific single-day output sequence curves are shown in Figure 14. The accuracy of the prediction model can be further improved by separately train the different categories of weather in the subsequent experiments.

As it can be seen, in the sunny day scenario, the PV output is smoother and the magnitude fluctuates around 100kW. In this scenario, the prediction accuracy is the highest compared to other scenarios due to the high periodicity, trend and seasonality of the data. In the cloudy day scenario, the PV output fluctuates greatly due to the uncertainty of cloudy and sunny conditions during the day. The highest value fluctuates around 80kW, the trend is obviously weakened, and the data characteristics are not obvious. The traditional forecast model in this scenario is difficult to capture the correlation between meteorology and PV, and the prediction is difficult. In the rainy day scenario, the PV output varies greatly due to the decrease in radiance and temperature. The highest value is around 50kW, and the trend is the least obvious. The meteorological data of rainy day itself is not regular, it is difficult to find the long-term dependence relationship with PV power, and the prediction difficulty reaches the maximum.

### TCN-Wpsformer model point forecast effect

**Fig. 15:**
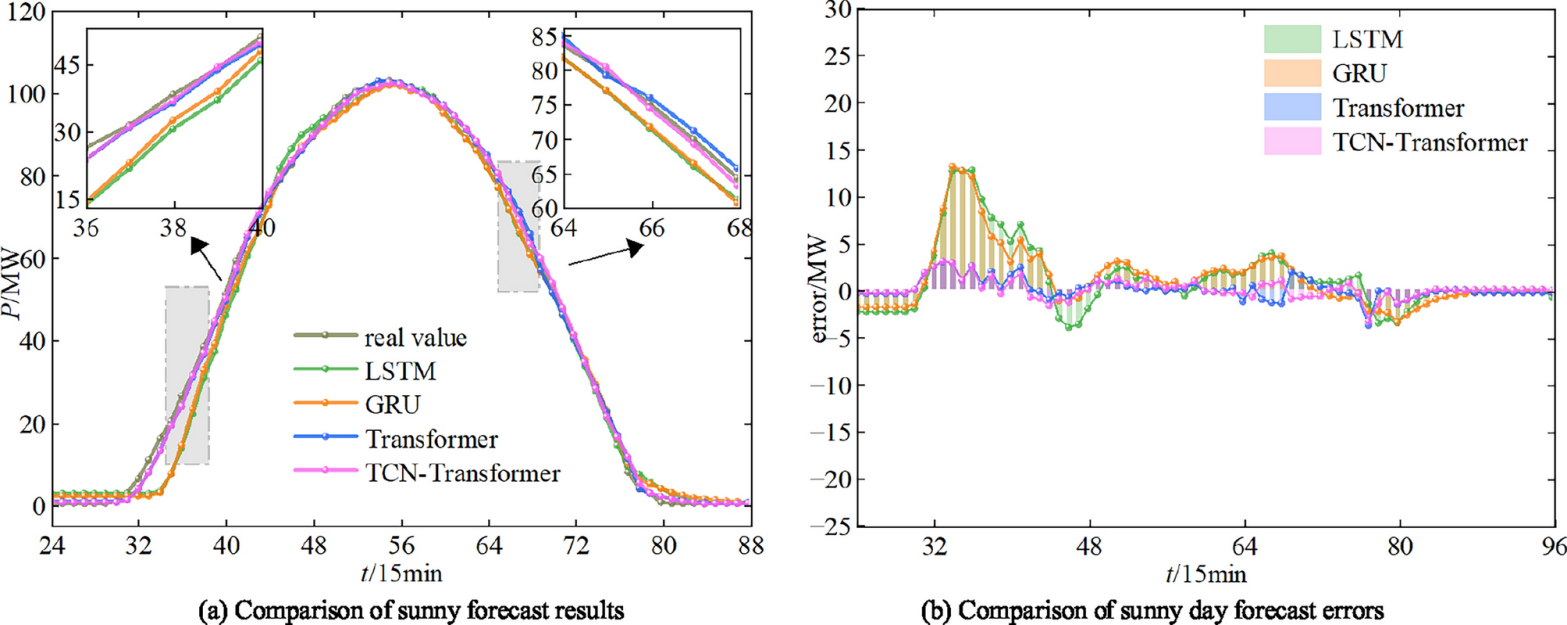
Comparison of predicted results of PV power on sunny day.

**Fig. 16:**
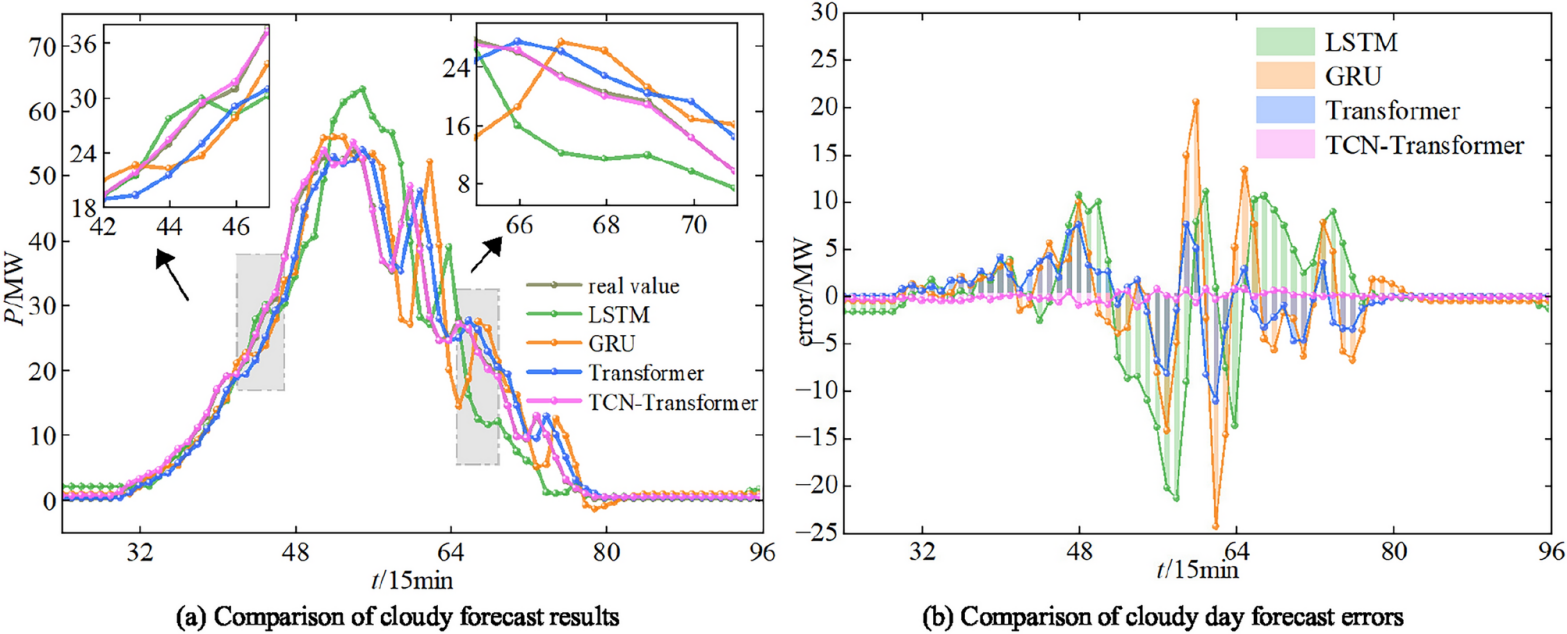
Comparison of PV power prediction results for cloudy day.

**Fig. 17:**
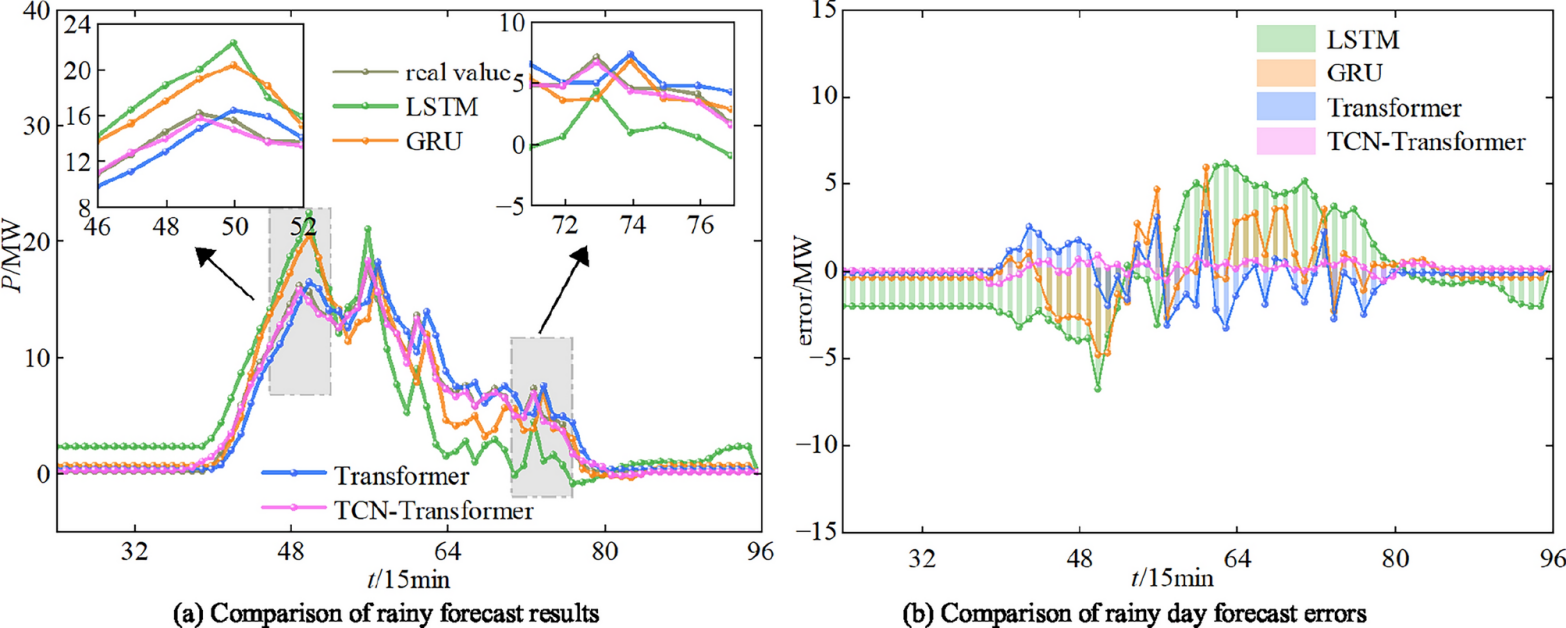
Comparison of PV power plot prediction results for rainy day.

The simulation environment used in this work is as follows. Windows 10 operating system, 32GBRAM, CPU is Intel Core i7-12400F @2.5G Hz, GPU is NVIDIA GeForce GTX 3060ti, and Python 3.7 language is used for the framework construction. In order to verify the effectiveness of the model proposed in this study in PV power forecast, the TCN-Wpsformer model proposed is compared with the LSTM model, the GRU model, and the original Transformer model. The PV power forecast results of the different models are shown in Figure 15, Figure 16, and Figure 17.

As it can be seen from Figure 15, the model proposed in this work can predict the degree of PV power fluctuation accurately in the period of power rise, fall and rapid fluctuation. While the traditional Transformer model, LSTM model and GRU model still have a certain lag in the process of prediction. In particular, the data in the sunny day scenario is smooth, fixed in magnitude, less volatile and least variable between similar days. Therefore, the prediction values of the four models can track the data fluctuation changes better. From the overall indicators, the TCN-Wpsformer prediction effect is closest to the real value, and the error indicators are all lower than the comparison model.

In the cloudy scenario, the sample data fluctuate drastically, with slightly lower amplitudes than the sunny scenario. The weather characteristics are unstable, and the periodicity and trend are weakened. The cloudy overcast sky reduces the correlation between irradiance and PV power, which weakens the long-term dependence between the long time series, thus leads to a decrease in the prediction accuracy. The error index is still the least in the TCN-Wpsformer model.

In the rainy day scenario, the data fluctuate more dramatically than in the sunny day scenario, and the amplitude is reduced to only about 10kW. All kinds of meteorological data changes greatly compared with the sunny day scenario. The dependence between various meteorological factors and PV power is weakened, and the characteristics of the changes are difficult to capture. It can be seen from the prediction curves that TCN-Wpsformer has a smaller error increment than the traditional prediction model. Table 3 shows the results of the comparison of the evaluation indicators of different models on different similar days.

**Table 3:** Comparison of evaluation indicators.

Similar day types	Model	Single-step forecast			Four-step forecast		
		$E_{\textrm{MSE}}$/ MW	$E_{\textrm{MAE}}$/ MW	$R^{2}$/ %	$E_{\textrm{MSE}}$/ MW	$E_{\textrm{MAE}}$/ MW	$R^{2}$/ %
sunny day	LSTM	3.3212	2.1624	98.5725	5.0548	3.3423	96.5636
	GRU	2.6542	1.6571	98.8293	4.1846	2.1411	97.9878
	Transformer	1.2841	0.8966	99.6258	1.3491	1.0017	99.4428
	TCN-Wpsformer	0.4111	0.3446	99.9390	0.4094	0.3735	99.7714
cloudy day	LSTM	2.2122	1.7433	97.1455	4.5549	2.9662	96.5696
	GRU	2.0284	1.4841	97.9612	4.2552	2.0784	97.1086
	Transformer	1.4141	1.1892	98.7977	1.5474	1.3419	98.1158
	TCN-Wpsformer	0.6149	0.7366	99.1394	0.5906	0.8749	98.8431
rainy day	LSTM	3.2712	2.3185	94.2127	4.7155	5.5771	93.5004
	GRU	1.4014	1.2452	97.2679	3.6383	3.4343	96.1179
	Transformer	0.3950	0.4422	98.5236	0.3645	1.4841	97.9484
	TCN-Wpsformer	0.3212	0.3461	98.6525	0.3077	0.4812	98.6299

In summary, meteorological data under cloudy and rainy weather scenarios are highly volatile, with weakened periodicity and trend. Traditional prediction models are difficult to capture the temporal characteristics in such scenarios. At this time, the TCN-Wpsformer model is improved to observe the data globally. Its multi-attention structure better captures the couple relationship between meteorological factors and PV power in time series with large data fluctuations. Compared to the original Transformer model, the $E_{\textrm{MAE}}$ and $E_{\textrm{MSE}}$ metrics reduced by 38.05% and 56.51%, for the cloudy day. The $E_{\textrm{MAE}}$ and $E_{\textrm{MSE}}$ metrics reduced by 21.69% and 18.68%, for the rainy day. Through the self-attention mechanism, the dependency between PV and meteorological data under conditions of drastic data changes is emphasized. Weakened couple relationship between meteorological data and PV power under cloudy and rainy scenarios. The multi-layer self-attention structure captures the weak feature relationships between different parts of multi-dimensional long time series. It also learns richer feature representations and higher prediction accuracy compared to traditional models in cloudy and rainy scenarios.

### TCN-Wpsformer model interval forecast effect

Point forecast results do not adequately capture the uncertainty in electricity loads, so it is critical to evaluate interval forecast results. In this study, interval forecast analysis is performed based on the use of point forecasts. The prediction interval coverage probability (EPICP) and prediction interval normalized average width (PINAW) are selected as the evaluation indexes for interval forecast.

22 $$\begin{aligned} E_{\textrm{PINAW}}= & \frac{1}{N}\sum _{i = 1}^{N}\frac{U_{i} - L_{i}}{y_{\textrm{max}} - y_{\textrm{min}}} \end{aligned}$$

23 $$\begin{aligned} E_{\textrm{PICP}}= & \frac{1}{N}\sum _{i = 1}^{N}K_{i}\ \end{aligned}$$

Where, $U_{i}$ is the upper boundary value for the ith sample. $L_{i}$ is the lower boundary value for the ith sample. $k_{i}$ is a binary variable that is noted as 1 if the actual power is within the model prediction interval and 0 otherwise.

**Fig. 18:**
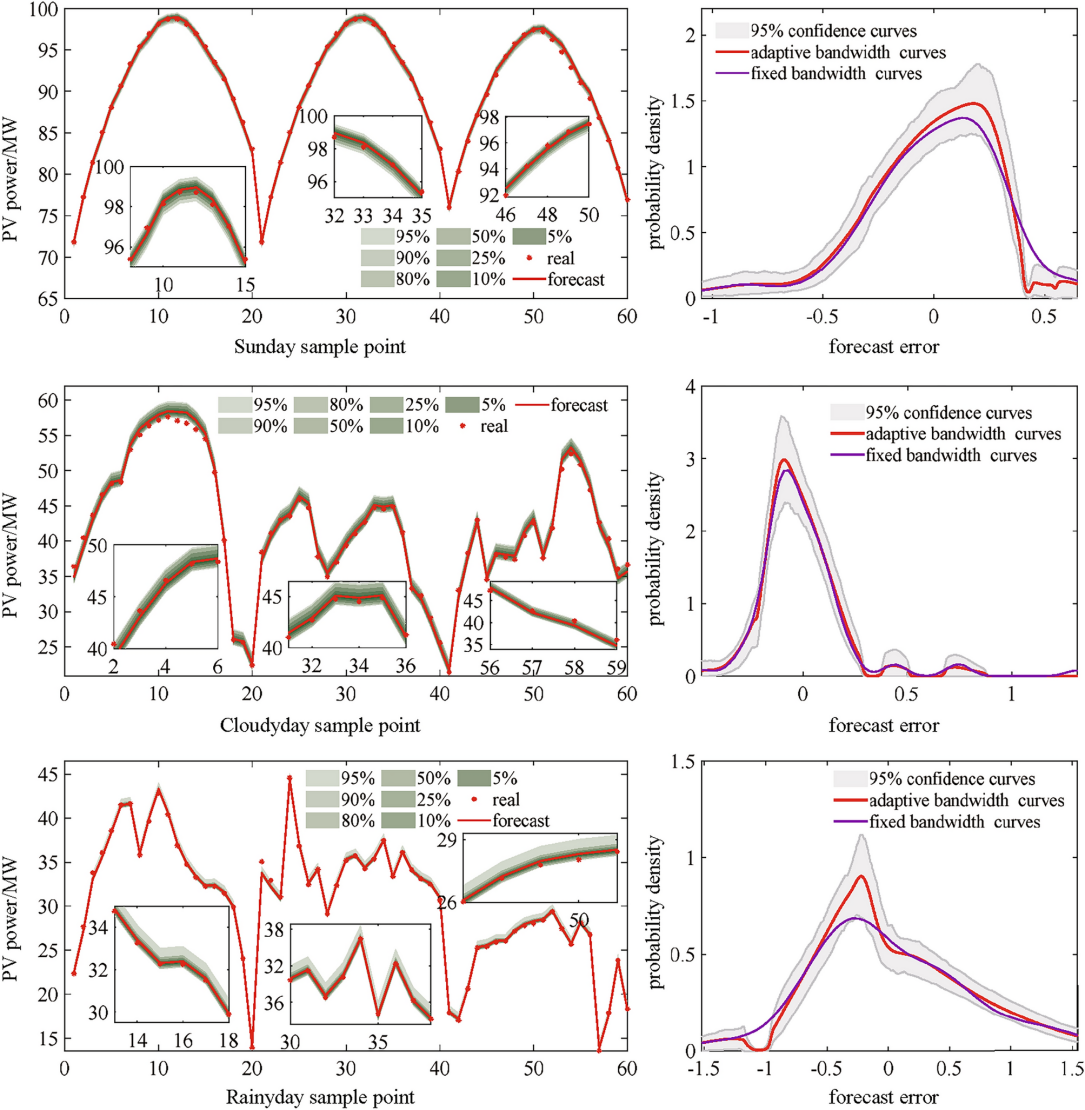
Comparison of PV power interval prediction results.

In order to obtain a more accurate prediction of PV intervals, a nonparametric method adaptive bandwidth kernel density estimation curves is selected, which fits the error between the measured and point prediction of PV power under each weather type. The kernel density estimation can be used to find the error range at different confidence levels. A corresponding interval prediction range is obtained with the point prediction results superimposed on the error range. The interval prediction results within the 5%~95% confidence interval are calculated for the three weather types separately. The three weather type sampling days are selected randomly from the data sample as a validation set for interval prediction by kernel density estimation. The evaluation results are shown in Table 4 and the interval prediction results are shown in Figure 18.

**Table 4:** Predictive performance for each confidence interval.

Similar day type	Evaluation indexes	Confidence interval				
		95%	90%	80%	50%	50%
sunny	PICP	0.9891	0.9472	0.9175	0.8895	0.7961
	PINAW	0.1134	0.0932	0.0726	0.0567	0.0396
rainy	PICP	0.9709	0.9306	0.9170	0.8313	0.7440
	PINAW	0.1039	0.0854	0.0666	0.0519	0.0350
cloudy	PICP	0.9857	0.9499	0.9242	0.8854	0.7604
	PINAW	0.0691	0.0568	0.0442	0.0350	0.0241

The prediction model proposed in this work has more than half of the predicted objects within the intervals when the confidence interval is 50% for all three weather types when interval prediction is performed. The PICP under turnaround weather is lower than that of sunny and rainy weather, which is a better adaptation to turnaround weather. Taken together, in the actual prediction, better interval prediction results can be achieved when the confidence level is taken from 95% to 80%. So, This model can give more accurate interval prediction results under the condition of no meteorological factor input. Besides, It can satisfy the problem of point prediction, which is difficult to quantify PV power generation in practical application, within the corresponding confidence level.

### Ablation experiment

Table 5 shows the details of hyperparameter settings for the TCN-Wpsformer PV power prediction model. It mainly consists three parts parameters which are TCN, Wpsformer and training strategy. The model parameter configuration can be adapted to the transparency needs of a particular power station. Some of the module and parameter effects on model performance performance can be locally assessed by ablation experiments. The description of design ablation experiment operation is shown in Table 6.

**Table 5:** Role and rationale of model parameters setting.

Parameters	Value	Role and rationale
Dilation rate	[1,2,4,8]	exponential growth and expanded sensory field
Numblocks	4	balance feature abstraction capabilities and gradient disappearance risk
Stride	1	keep output length consistent with input to avoid loss of key time point information due to step >1
Filtersize	4	convolution kernel covers 1 hour at 4 time steps
Numfilters	32	32 convolutional kernels to extract multi-scale temporal features
Paddingsize	[8, 0]	left/right padding steps to ensure causal convolution does not leak future information
Numheads	3/4	multi-head mechanisms focus on different scale features
Windowsize	1/4	corresponds to 15min/1 hour hour time span
Dropout probability	0	no dropout, as the L2 regularisation of TCN (1e-4) provides sufficient generalisation
Sparsity threshold	0.3	dynamically block time steps with attention weights below 0.3 to reduce redundant computation
Initial learningrate	0.1	accelerate initial convergence with segmental descent to adapt to nonlinear changes in irradiance
L2 regularisation factor	1e-4	constrain TCN convolutional kernel weight smoothness and suppress data high-frequency oscillations
Minibatchsize	96	single batch covers one day data and ensures the contextual integrity of causal convolutions
Maxepochs	100	provide a buffer for the early stop mechanism to avoid misjudging convergence due to short-term fluctuations
Validationpatience	10	If the validation loss does not improve over 10 consecutive epochs, the training is terminated early

**Table 6:** Specific descriptions of ablation experiments.

Operating objects	Operation description	MAE/MW	R2/%	Computing time/s
Model component	TCN+Wpsformer+FCM	0.2177	99.9721	127.8079
	Wpsformer+FCM	1.2588	98.4848	104.4502
	TCN+FCM	1.6454	98.2011	324.3255
	TCN+Wpsformer	0.6838	99.1466	116.2423
L2 regularisation coefficient	For 1e-4 read 0	0.2946	99.5568	113.4845
Sparsity Threshold	From 0.3 to 0.1	0.3289	99.2779	111.0045
Initial learning rates	From 0.1 to 0.05	0.2632	99.9385	140.2811

From the experiments on model component removal, it can be concluded that FCM preprocessing improves the model prediction accuracy by working condition classification. Wpsformer+TCN effectively captures the irradiance decay trend. Compared with removing a single component, the complete model TCN+Wpsformer+FCM has the highest prediction accuracy. From the experiments on parameter adjustment, it can be seen that L2 regularisation effectively suppresses the overfitting caused by sensor noise. Too low a sparse threshold loses the attentional weights of the critical time step, which leads to a decrease in accuracy. In addition to this, a higher initial learning rate accelerates the convergence of TCN shallow features.

### Model complexity and interpretability

**Fig. 19:**
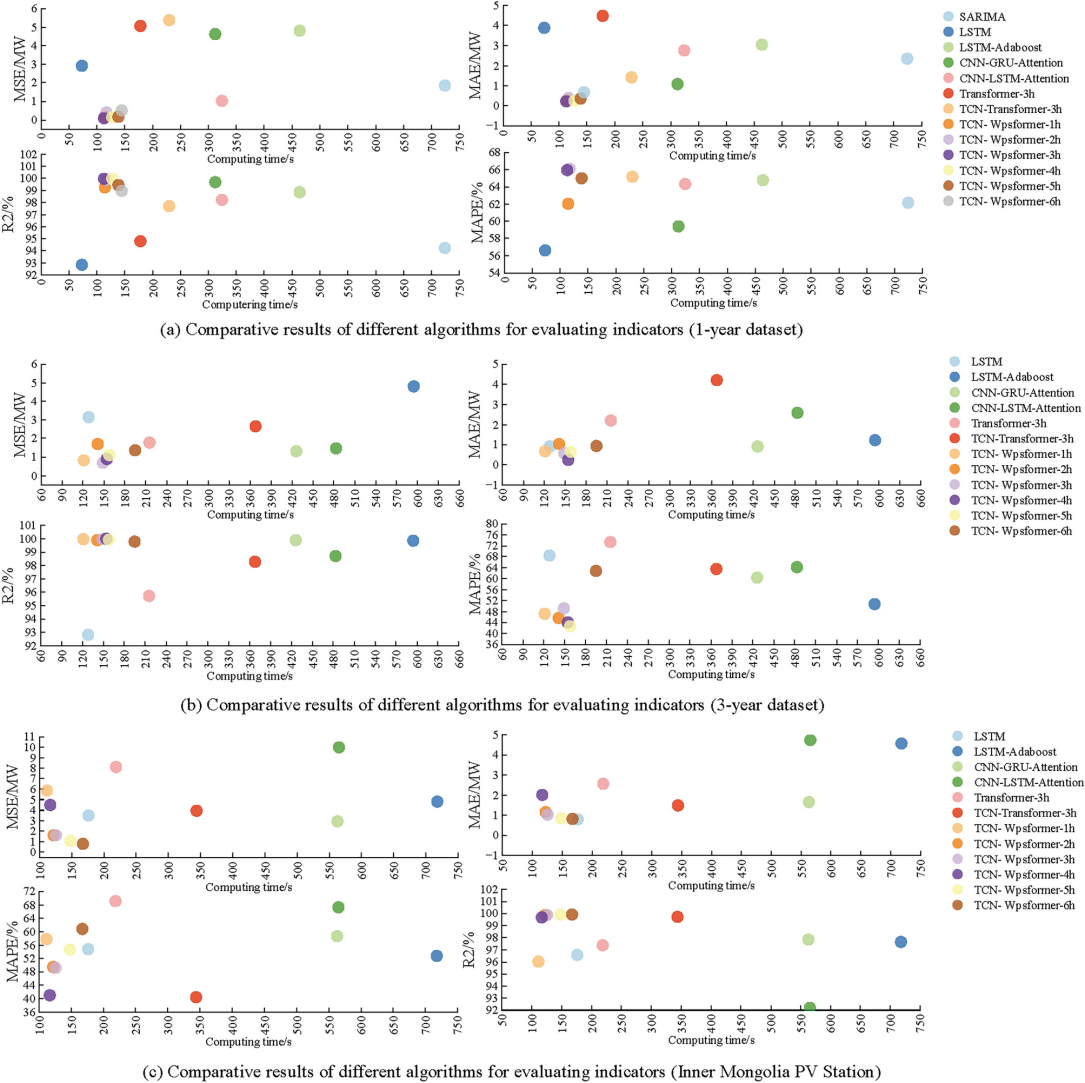
Comparison plot of computing complexity and accuracy.

In order to show the performance comparison of the models in different data amount and different power station , the continuous 1 year and 3 years data of a PV power station in Gansu and the continuous 5 years (2016~2020) data of a PV station in Inner Mongolia are selected for the compare. The contrast models are SARIMA, LSTM, LSTM-Adaboost, CNN-LSTM-Attention, CNN-GRU-Attention, Transformer-3h, TCN-Transformer-3h and the number of this research/attention head is 1~6. The prediction steps are all 4 steps for 1h. The scatterplot is plotted with the algorithm computing time as the horizontal axis and the evaluation metrics MSE, MAE, MAPE and R2 as the vertical axis, and the results are shown in Figure 19.

From Figure 19(a) and Figure 19(b), it can be seen that the Transformer class algorithms have high overall prediction accuracy and better running time than the SARIMA and LSTM class models. The difference in the number of attention heads also affects the prediction results. 1-year dataset are all TCN-Wpsformer-3h with the shortest computation time and the largest R2. 3-year dataset are TCN-Wpsformer-1h with the shortest computation time and TCN-Wpsformer-4h with the largest R2. Taking the evaluation index MAE as an example, the TCN-Wpsformer-3h method is the most effective in the 1-year dataset, with a computation time of only 112 s. Compared with Transformer, the accuracy is improved by 5.45%, while the time is reduced by about 66 s. This is due to the introduction of the window sparse self-attention. This is due to the introduction of window sparse self-attention, which can reduce the original computational complexity $O(n^{2})$ to *O*(*nw*) , and the effect is especially significant with the increase of sequence length. For example, the computation time of TCN-Wpsformer-3h in the 3-year dataset is only 1.32 times that of the 1-year dataset. This compares to 1.61 times for TCN-Transformer-3h and 1.49 times for CNN-LSTM-Attention.

**Fig. 20:**
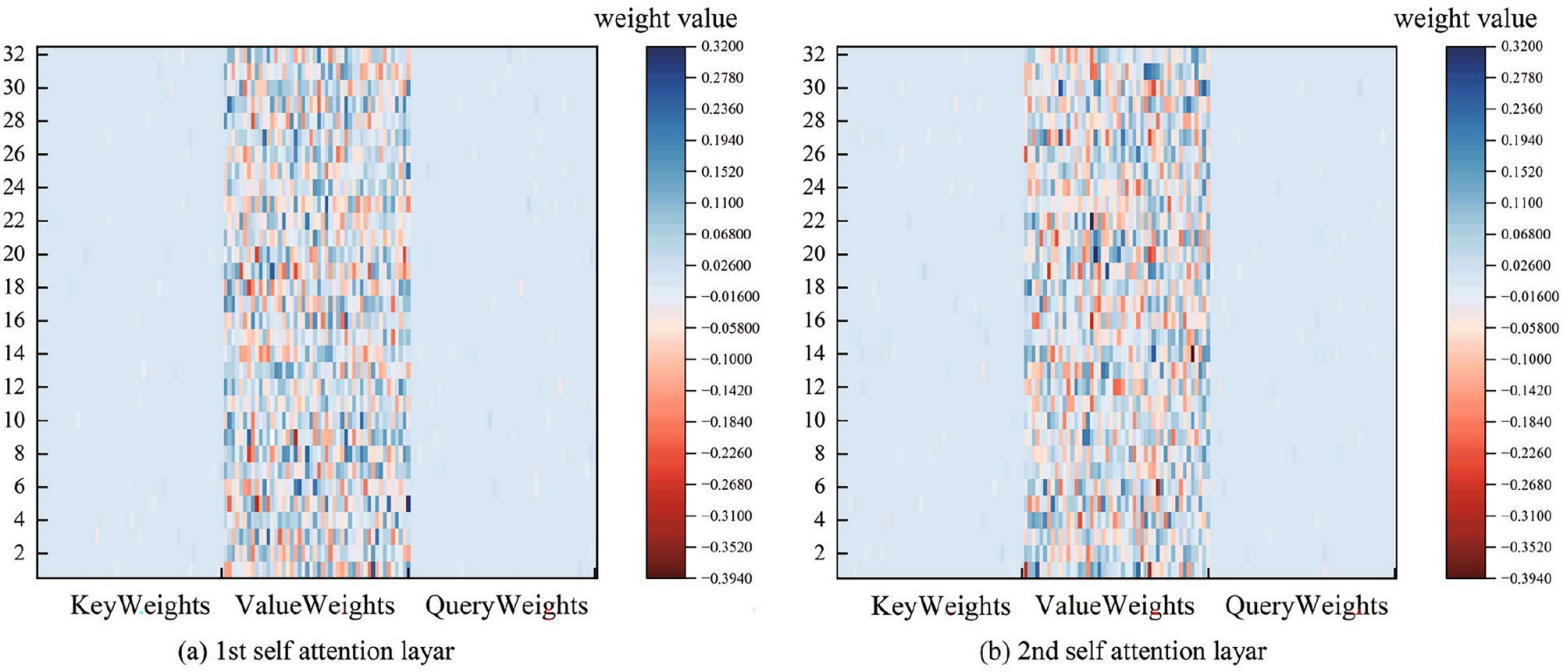
Attention mechanism ***KQV*** weights matrix heat map.

In response to operators’ preference for transparent models, the heat map of the weight matrix of the attention mechanism can be used to promote the evolution of PV prediction models from the black box to the white box, and to enhance their credibility in power system scheduling. Figure 20 shows the heat map of the ***KQV*** weight matrix of the proposed model.

The weight heat map visualisation shows that: the ***KQ*** matrix shows a diagonally strong response, which is due because they jointly determine the attention score ${\varvec{{QK}}}^{T}$. this phenomenon suggests that the PV power prediction model pays more attention to correlations at the same locations in the input sequence. the ***V*** matrix weights are more evenly distributed, suggesting that the model retains the multidimensional raw information in case of overreliance on a specific feature. This visualisation provides an intuitive basis for understanding the internal mechanisms of Wpsformer and somewhat avoids black-boxed hybrid feature interactions.

## Conclusion and outlook

In order to solve the problem that the gradient descent-based RNN is difficult to capture the long-term dependence of PV power with long time span, and the current Transformer model is unable to follow the rise or fall of PV power quickly. A hybrid model based on TCN-Wpsformer for short-term PV power prediction is proposed in this paper, which is composed of three main components.

1. The Pearson interpolation PV data restoration method and PCA-FCM data similar day clustering method are used. It can divide the PV output sequence into sunny, cloudy and rainy datasets. By ablation experiments, it is demonstrated that the addition of data repair clustering session $E_{\textrm{MAE}}$ can effectively improve 0.4661 MW, $R^{2}$ by 1.77%. It indicates that data preprocessing can improve the model adaptability to different weather scenarios through work condition classification. The data preprocessing output can be directly mapped to weather-equipment state labels familiar to operators and maintainers. It provides physically interpretable groupings of inputs for subsequent models.
2. TCN is introduced ahead of the self-concerned mechanism. In this way, the linkage between time segments can be established and the coupling between climate factors and PV power can be better captured. Compared to the original Transformer model, the $E_{\textrm{MAE}}$ , $E_{\textrm{MSE}}$, and $E_{\textrm{MAPE}}$ metrics by 61.29%, 71.24%, and 32.95%, respectively, for the 3 year dataset. Removing the TCN module decreases the $E_{\textrm{MAE}}$ prediction accuracy by 1.0411 MW, which indicates that the TCN captures the long-term dependence between the splicing matrices. It can achieve the purpose of accurately capture the trend of irradiance change.
3. Finally, the window probability sparse self-attention mechanism is proposed. It can reduce the computational burden of product correlation within each observation point. As for the speed of model training, the TCN-Wpsformer model takes 148s while the original Transformer model takes 215s and performs better in terms of prediction accuracy. A visual analysis of the attentional mechanism weight matrix provides an intuitive basis on which the model digs deeper into the data features, to a certain extent avoiding the black-boxed mixture of feature interactions.

The proposed method is particularly suitable for large and fast time series data. It is due to the fact that Wpsformer is based on the Transformer improvement, which reduces its computational complexity from $O(n^{2})$ to *O*(*nw*) . The model can be adapted to more data types by extending input dimensions through a parallel branch structure. For example, when new weather radar data is added, it is only necessary to add the corresponding feature embedding layer in the Wpsformer module without reconstructing the overall architecturewhich can cope with large-scale PV power plant cluster data. In order to meet the real-time scheduling requirements of power grid operation, the long-term pattern learning of FCM clustering and Wpsformer is completed in the offline phase, namely, it is updated once a day. The online prediction only needs to call the lightweight TCN module and pre-stored attention templates, which reduces the real-time prediction time to 72.23% of the traditional Transformer model.

Although the combined model proposed in this study performs better than the comparison model in terms of PV power prediction accuracy, and model computing timeliness. However, there are still limitations in terms of data dependence and model complexity. In the future, the design of robust data interpolation strategy or the introduction of uncertainty estimation module can be considered to alleviate this problem. For example, the novel ramping behavior analysis (RBA)^40^ technique compresses the number of parameters to adapt to edge computing devices for model lightweighting purposes. In addition, the deep nonlinear structure of TCN and Wpsformer leads to the prediction results are difficult to be interpreted intuitively, which is not conducive to the power dispatchers’ understanding of the model decision logic. It can be partially mitigated by attention weight heatmap visualisation, but still needs to be combined with physical relationships to enhance interpretability.

## Data Availability

Available on reasonable request from the corresponding author.
